# Biology and epidemiology of *Diaporthe amygdali*: understanding how environmental factors influence fungal growth, sporulation, infection and lesion development on almond

**DOI:** 10.3389/fpls.2025.1717223

**Published:** 2025-12-10

**Authors:** Carolina Francia, Elena Lázaro, Maite Novellón, Antonio Ramón-Albalat, Francisco Beluzán, Antonio Vicent, Mónica Berbegal, Josep Armengol

**Affiliations:** 1 Instituto Nacional de Investigación Agropecuaria, INIA La Estanzuela, Colonia, Uruguay; 2 Instituto Agroforestal Mediterráneo, Universitat Politècnica de València, València, Spain; 3 Departament d’Estadística i Investigació Operativa, Universitat de València, Burjassot, València, Spain; 4 Centre de Protecció Vegetal i Biotecnología, Institut Valencià d’Investigacions Agràries, Moncada, Valencia, Spain; 5 Instituto de Investigaciones Agropecuarias, INIA Carrillanca, Temuco, Chile

**Keywords:** epidemiological modeling, hierarchical additive models, nut crops, *Prunus dulcis*, temperature, twig canker and shoot blight, wetness period

## Abstract

**Introduction:**

*Diaporthe amygdali* is a major pathogen causing twig canker and shoot blight disease on almond crops. Knowledge about the influence of environmental factors on the biology and epidemiology of this pathogen has mainly been obtained on peach, but there is scarce information on almond. Thus, the main objective of this research was to better understand how environmental factors, such as temperature and wetness periods, determine the cycle of this disease on almond crops in Mediterranean conditions.

**Methods:**

Several experiments were conducted to obtain information about mycelial growth, sporulation, plant infection and lesion development using almond isolates of *D. amygdali*.

**Results:**

Our results showed that the temperature ranges for both α-conidia germination and almond infection are broad and overlap (between 5 and 35°C), potentially allowing infections to occur year-round. Nevertheless, the highest infection of almond plants was observed after 72 h of wetness period, while the lowest occurred after 6 h, explaining why *D. amygdali* is a prevalent disease in spring and autumn when rain events are more frequent.

**Discussion:**

Mycelial growth of *D. amygdali* and lesion development were promoted by warm temperatures. The production of mature pycnidia on cankers had lower and narrower temperature requirements, thus suggesting an adaptation to late winter and early spring conditions. Moreover, the optimum temperature for α-conidia production in pycnidia was around 22°C. All this information could be used to develop a mechanistic model for almond twig canker and shoot blight disease management to enhance the timeliness and effectiveness of control strategies, while reducing both economic costs and environmental impacts.

## 1 Introduction

The genus *Diaporthe*, includes plant pathogens, non-pathogenic endophytes and saprobes, being currently composed by nearly 1200 described taxa (Rossman et al., 2007; Gomes et al., 2013; Dissanayake et al., 2024). *Diaporthe* species have been associated with several important plant diseases worldwide affecting many economically important crops (Uecker, 1988; Udayanga et al., 2011; Gomes et al., 2013; Huang et al., 2013).

Within phytopathogenic *Diaporthe* species, *D. amygdali* has been reported as associated with canker diseases, also named “constriction cankers”, in a wide range of woody crops such as almond (Adaskaveg et al., 1999), apricot (Garofalo, 1973), blueberry (Hilário et al., 2022), chestnut (Aghayeva et al., 2018), grapevine (Van Niekerk et al., 2005), nectarine (Beluzán et al., 2021), papaya (Alam et al., 2023), peach (Brugneti et al., 2025), pear (Bai et al., 2015) and walnut (López-Moral et al., 2020a; Meng et al., 2018), in different regions worldwide. In the Mediterranean Basin, *D. amygdali* is a major pathogen causing twig canker and shoot blight disease on almond crop, which has been reported in Croatia (Župić and Kožarić-Silov, 2017), Hungary (Varjas et al., 2017b), Italy (Gusella et al., 2023), Portugal (Diogo et al., 2010), Spain (Tuset and Portilla, 1989; León et al., 2020) and Tunisia (Trigui, 1968), being especially prevalent in Spain (León et al., 2020).

Symptoms caused by *D. amygdali* on almond trees include necrotic and brown lesions (1 to 5 cm in diameter), initially formed around buds on green shoots, which further develop into annual, nodal and sunken cankers, sometimes with a little gummy exudate, as well as the withering of twigs. As a result, leaves wilt and, when the disease is severe, defoliation can occur and it can also affect flowers due to desiccation (Tuset and Portilla, 1989; Batlle et al., 2017; Varjas et al., 2017a).

*Diaporthe amygdali* produces pycnidia on twig cankers, which are globose or applanate, sometimes ellipsoidal, dark brown to black, measuring between 180 and 500 µm in diameter. Mature pycnidia exude cirri of α-conidia through their ostioles (Tuset and Portilla, 1989), which are hyaline, fusiform, straight with slightly acuminate ends, unicellular and measure from 4.9 to 9.9 µm in length by 2.3 to 3.8 µm in width, while β-conidia are seldom produced (Tuset and Portilla, 1989; Adaskaveg et al., 1999; Lalancette and Robison, 2001; Meng et al., 2018). It is known that conidia of *D. amygdali* infect leaves, twigs and fruit through leaf scars in autumn and through buds, bud scale scars, blossoms, and fruit scars in spring (Tuset and Portilla, 1989; Lalancette and Robison, 2001). In USA, it was described that on peach crop the duration of the incubation period, from infection to first canker symptoms, is approximately one month (Guba, 1958).

The management of *D. amygdali* in almond orchards relies on calendar-based phytosanitary treatments, adjusted according to phenological stages of the crop (Lalancette and Robison, 2002; Arquero et al., 2013). However, the use of fungicides is limited due to the scarcity of authorized active ingredients (Arquero et al., 2013; Gil et al., 2015). Recommended treatments are mainly preventive and applied prior to flowering and leaf fall periods, based on the assessed orchard risk and local weather conditions (Gil et al., 2015). For new plantings, it is recommended to consider the varying susceptibility to *D. amygdali* of different almond cultivars (Beluzán et al., 2022). Removal of affected twigs through summer and winter pruning should be done to reduce sources of inoculum (Gil et al., 2015).

The effect of temperature and other environmental factors on mycelial growth, pycnidia and conidia development, as well as infection, has been studied in detail for several *Diaporthe* species such as *D. citri* on citrus (Mondal et al., 2007), *D. eres* on hazelnut (Arciuolo et al., 2021), *D. neotheicola* on pistachio (López-Moral et al., 2020b) and walnut (López-Moral et al., 2022), and *D. phaseolorum* on soybean (Grijalba and del C Ridao, 2014). Regarding *D. amygdali*, most studies have been conducted on peach or using peach isolates. For instance, Zehr (1995) described some aspects of the biology of *D. amygdali* on peach in relationship with the temperature. These authors reported that α-conidia germinate on moist surfaces between 5 and 36°C with an optimum of 27 to 29°C. These authors also stated that infection is most common at 5 to 15°C. In addition, in USA pycnidia development was studied by Lalancette and Robison (2001) in both field and laboratory conditions. These authors indicated that the size of cankers and the number of pycnidia per canker of *D. amygdali* from peach followed a sinusoidal pattern, with lowest values in late winter and early spring and maximum values in late summer and early autumn. Lalancette et al. (2003) evaluated cirri production per pycnidium and conidia per canker. In this study, cirri production was observed between 1 and 38°C, with the greatest proportion of pycnidia producing cirri at 17°C, and the optimal production of conidia per pycnidium was slightly warmer at 24°C. In Brazil, *D. amygdali* isolates collected from peach showed an optimum mycelial growth at 25°C (Corrêa, 2014). In Italy, Brugneti et al. (2025) found an optimum mycelial growth of *D. amygdali* isolates obtained from symptomatic peach trees around 24°C. In this study, the maximum and minimum temperature thresholds were 40°C, and between 5°C and 7°C, respectively. Despite the available information obtained from peach *D. amygdali* isolates, the influence of environmental factors on the biology of this pathogen on almond trees is still largely unknown, with only two studies that partially addressed this issue. Gusella et al. (2023) evaluated the effect of temperature on mycelial growth and lesion development of some almond *D. amygdali* isolates collected in Sicily (Italy). These authors observed that mycelial growth *in vitro* occurred between 5 and 30°C with an optimum of 25°C, and lesions development on detached shoots occurred between 5 and 35°C with an optimum of 20.8°C. Beluzán et al. (2025) observed that the availability and dispersal of *D. amygdali* inoculum to nearby branches and trees in almond orchards in Spain are significantly influenced by weather variables such as temperature range, relative humidity, rainfall, and wind speed.

In addition to the environmental factors, quantitative traits of pathogenicity vary with the host and the pathogen genotypes, and their interaction (Lannou, 2012). This emphasizes the importance of studying the effect of environmental factors on the biology of *D. amygdali* using almond isolates and almond plants. As Lannou (2012) indicated, measuring quantitative traits is essential to get information about disease dynamics and pathogen evolution. Decomposing the pathogen cycle into elementary life traits allows getting information to develop epidemiological models. These models represent the basis of most decision tools in integrated pest management programs, to enhance the effectiveness and efficiency of control strategies (Rossi et al., 2019).

Thus, our work aims to obtain new information about the effects of environmental factors on the growth, sporulation, infection and lesion development of *D. amygdali* on almond. These traits are critical for epidemiological modeling because they directly explain how the pathogen grows, spreads, and causes new infections. Several experiments were conducted with the following objectives: i) to determine mycelial growth, pycnidia development, and α-conidia production at different temperatures; ii) to evaluate α-conidia germination at different temperatures and periods of incubation; iii) to study the lesion development on detached almond twigs at different temperatures; and iv) to evaluate the infection of almond plants at different temperatures and wetness periods.

## 2 Materials and methods

### 2.1 Fungal isolates

Two fungal isolates of *D. amygdali*, PHAL-4 and PHAL-45, obtained from almond trees showing symptoms of twig canker and shoot blight disease in Spain, which were characterized previously (León et al., 2020; Hilário et al., 2021), were used. These isolates were stored in 15% glycerol solution and stored at –80°C in 1.5-ml cryovials in the fungal collection of the Instituto Agroforestal Mediterráneo – Universitat Politècnica de València (Spain). They were grown on potato dextrose agar (PDA; Biokar-Diagnostics, Zac de Ther, France) for 7 days at 25°C in the dark prior to experimental assays.

### 2.2 Mycelial growth, pycnidia development and α-conidia production at different temperatures

Seven temperatures (5, 10, 15, 20, 25, 30 and 35°C) were evaluated in this experiment. For mycelial growth, mycelial plugs of isolates PHAL-4 and PHAL-45 were obtained from the margin of actively growing colonies using a sterile 6.0 mm-diameter cork borer and were plated in the center of PDA plates. The plates were not sealed and then were incubated at the temperatures indicated above with a photoperiod of 12 h light and 12 h darkness for 21 days. Treatment replicates (applying the same treatment across multiple experimental units) consisted of three Petri plates per isolate and temperature combination arranged in a completely randomized design. Two perpendicular diameters were measured in each colony every 3 days and the average of the two diameters was converted to radial growth rate (mm day^-1^). The Petri plates used for the mycelial growth study at different temperatures were also observed for the formation of mature pycnidia (with presence of cirri of α-conidia) and to determine the number of α-conidia produced per pycnidium. For this purpose, after 21 days of incubation, photographs of the colonies were taken and the numbers of mature pycnidia were visually quantified. These were related to the total surface of the plate, obtaining the total number of mature pycnidia per colony. Subsequently, each plate was washed with 12 ml of sterile water and from the resulting suspension, the α-conidia/ml were counted 4 times per plate in a Neubauer chamber, calculating then the number of α-conidia per pycnidium. This experiment was performed with four experimental replicates, consisting of two replicates per isolate (experimental replication = repeating the entire experiment in different times or locations).

### 2.3 Development of mature pycnidia on almond twigs at different temperatures and days post-inoculation

The production of mature pycnidia on the surface of almond twigs was evaluated at four temperatures: 10, 15, 20 and 25°C and at 7, 12, 17, 22, 27, 32, 37, 42 and 47 days post-inoculation. For this purpose, autoclaved segments of almond twigs of the susceptible cv. ‘Lauranne Avijor’, about 5 mm in diameter and 4 cm in length, were embedded for 12 h into α-conidial suspensions of *D. amygdali* isolates PHAL-4 and PHAL-45. These suspensions were obtained by washing cultures incubated at 25°C with a photoperiod of 12 h light and 12 h darkness for 30 days, with 12 ml of sterile water. The resulting conidial suspension was filtered through two layers of cheese cloth and was adjusted to 10^5^ α-conidia/ml using a Neubauer chamber. After inoculation, twigs were air-dried for 12 h in a laminar flow cabinet. Three air-dried twigs were placed on a plate with Water-Agar (WA: agar, 15 g; double distilled water, 1L) medium and incubated for 50 days at the temperatures indicated above with a photoperiod of 12 h light and 12 h darkness. Treatment replicates consisted of six Petri plates with three twigs per isolate and temperature combination. The production of mature pycnidia (with presence of conidial cirri of α-conidia) per twig segment was assessed approximately every 5 days from day 7 and subsequently rated on a qualitative 0 to 5 scale, where 0= none, 1 = 1 to 50, 2 = 51 to 100, 3 = 101 to 150, 4 = 151 to 200, and 5 = >200 pycnidia (Mondal et al., 2007). This experiment was performed with four experimental replicates, consisting of two replicates per isolate.

### 2.4 Conidia germination at different temperatures and periods of incubation

The germination of α-conidia of *D. amygdali*, isolates PHAL-4 and PHAL-45, was evaluated at seven temperatures (5, 10, 15, 20, 25, 30, and 35°C) and six incubation periods (6, 12, 18, 24, 36 and 48 h). Each treatment replicate consisted of four drops of 30 µl of conidial suspensions per combination of isolate, temperature and incubation period. The conidial suspensions were prepared as described above and adjusted at 5 x 10^5^ α-conidia/ml, and subsequently deposited on WA plates in incubators under dark conditions. Each drop was deposited on a separate WA plate. At the end of each incubation period, the percentage of germinated α-conidia was determined by examining 200 conidia on each drop by using a microscope (x40 magnification). A conidium was considered germinated when the length of the germinative tube was longer than the length of the conidium. This experiment was performed with four experimental replicates, consisting of two replicates per isolate.

### 2.5 Almond infection at different temperatures and wetness periods

Infection of *D. amygdali* on almond seedlings using isolates PHAL-4 and PHAL-45 was evaluated at seven temperatures (5, 10, 15, 20, 25, 30, and 35°C) and five wetness periods (6, 12, 24, 48, and 72 h). One-year-old seedlings of cv. ‘Lauranne Avijor’, 30 cm tall and growing on their own roots, were used. Five wounds were made on the stem of each plant using a hypodermic needle. First puncture was done on the main stem 5 cm over the surface of the substrate, and four additional punctures were done up in the stem with a separation of 3 cm each. A uniform layer of fine droplets of a conidial suspension of each isolate, prepared as described above and adjusted at 1x10^5^ conidia/ml, was gently sprayed on leaves and stems until runoff using a hand sprayer (W560, Wanger Spraytech Iberica S.A., Spain). Following inoculation, plants were covered with plastic bags to ensure continuous wetness and placed for incubation in growth chambers at the different temperatures in darkness. Each treatment replicate consisted of five random plants per unique combination of isolate, temperature, and wetness period. These five random plants were removed from the chambers after each wetness period was completed and surface dried by placing them 90 cm apart from a slow speed fan. Then, the plants were transferred to a growth chamber for incubation under suitable conditions for disease development (23°C; 60% RH; 12 h light/12 h darkness) in a randomized design. The infection was evaluated at 24, 48 and 72 h of incubation, determining the number of leaves with lesions and the number of leaves per plant. These values were then used to calculate disease incidence on leaves (%) for each combination of temperature (°C) and wetness period (h) as:


$$\text{Incidence on leaves }(\%)=\;\left[(\text{Leaves with lesion}/\text{total leaves per plant}{)}^{*}100\right]$$

Fragments of the inoculated stems were plated on PDA plates with 0.5 g/L of streptomycin sulphate (PDAS) to confirm by observing the morphology of the colonies that *D. amygdali* had infected the wounds. The number of infected wounds was used to calculate disease incidence on stems (%) for each combination of temperature and wetness period as:


$$\text{Incidence on stems }(\%)=\left[(\text{Infected wounds}/\text{total wounds per plant}{)}^{*}100\right]$$

This experiment was performed with four experimental replicates, consisting of two replicates per isolate.

### 2.6 Lesion development on detached almond twigs at different temperatures

The study of *D. amygdali* lesion development at different temperatures was carried out using 30-cm-long non-lignified detached almond twigs of the susceptible cv. ‘Vairo’ as described by Beluzán et al. (2022). The twigs were surface disinfected by dipping them in 70% ethanol for 30s, in 1.5% sodium hypochlorite solution for 1 min and finally in ethanol 70% for 30 s. Then, they were washed in sterile water and dried in a laminal flow cabinet. The twigs were wounded using a sterile cork borer (5.0 mm-diameter), the ends were re-cut to ensure water uptake, and then, immediately inoculated with a mycelial plug (5.0 mm-diameter) obtained from 15-day-old colonies of *D. amygdali* isolates PHAL-4 and PHAL-45 grown on PDA, which were inserted upside down into each wound. Finally, the lesion was sealed with Parafilm^®^. The inoculated twigs were covered with a plastic bag and kept for 7 days in an upright position with the base in a 1 L jar with 500 ml of sterile water and incubated at different temperatures (5, 10, 15, 20, 25 and 30°C) with 12 h light/12 h darkness. Each treatment replicate consisted of six twigs per isolate and temperature combination. Additionally, the control treatment was prepared with sterile PDA plugs. The lesion length (mm) of each twig was measured 15 days after the inoculation and mean lesion lengths were converted to lesion growth rate (mm day^-1^). This experiment was performed with four experimental replicates, consisting of two replicates per isolate.

### 2.7 Data analysis

For each experiment, the response variables and their relationships with the covariate(s) were examined through an exploratory analysis. Because of the non-linear patterns consistently identified across all experiments during this preliminary data analysis, the modeling approach was based on hierarchical generalized additive models (HGAMs; Wood, 2017; Pedersen et al., 2019). The complexity of the covariate space was addressed by incorporating smooth functions to capture non-linear relationships between the covariates and the response variable, as well as potential interactions in experiments involving two predictors. In addition, different random-effects structures were tested to account for potential hierarchical dependencies in the data, such as variability among experimental replicates. All models were fitted using R software (R R Core Team, 2025, version 4.2.2) and package *mgcv* version 1.9–1 (Wood, 2011).

For models where the response variable was associated with a single covariate (with X representing the covariate), the smooth function f(X) was specified using thin plate regression splines (TPRS; Wood, 2003), with the basis size (k) determined according to the heuristic approach: the number of levels of the covariate minus 2. The random effect smooth function (r()) was specified considering the default random smoother implemented in the *mgcv* package. Three random effects structures were explored: i) random intercept (r(Replicate)), allowing variation in the baseline level (intercept) of the response across experimental replicates, ii) random smooth (r(X, Replicate)), allowing the smooth effect of the covariate to vary by replicate, and iii) combined random intercept and random smooth (r(Replicate) + r(X, Replicate)) to model both the baseline differences and varying effects of the covariate across replicates simultaneously.

For models where the response variable was influenced by two covariates (X_1_ and X_2_), a two-dimensional smooth function (f(X_1_,X_2_)) representing their interaction was used. This smooth function was constructed as a tensor product smooth, combining marginal smoothers for each covariate. TPRS were applied to each covariate, with the basis size (k) determined according to the same rule of thumb described above. Four random effects structures were explored: i) random intercept (r(Replicate)) to account for variability in baseline response across experimental replicates, ii) random smooths for each covariate (r(X_1,_ Replicate) and r(X_2_, Replicate)), allowing the effect of each covariate to vary across replicates, iii) combined random intercept and random smooths for both covariates (r(Replicate) + r(X_1_, Replicate) + r(X_2_, Replicate)), modeling both the baseline differences and varying covariate effects across replicates simultaneously, and iv) random interaction effects (r(X_1_, X_2_, Replicate)), capturing variation in the joint effect of both covariates on the response across replicates. Analogous to the single-covariate case described above, from a biological point of view these structures provide alternative ways of capturing variability between experimental replicates. The random intercept accounts for differences in initial response levels, the random smooth allow replicate-specific variation in responses to each covariate, the combined structure accounts for both baseline and covariate-specific deviations simultaneously, and the random interaction captures replicate-specific variation in the joint effect of both covariates. These differences, captured in distinct ways by the different random-effect structures, may reflect normal experimental fluctuations and inherent isolate-related effects.

Detailed model specifications for each experiment and response variable, including the probability distribution of the response variable, the link function relating the mean response to the linear predictor, the covariate structure, and the random effects, are provided in **Supplementary Table 1**.

Each model was evaluated based on the Akaike Information Criterion (AIC) (Sakamoto et al., 1986), adjusted R^2^, deviance, and the number of parameters. Deviance was considered to assess the overall goodness-of-fit, while adjusted R^2^ measured the proportion of variability explained by the model, accounting for the number of parameters. However, the AIC was the metric considered for model selection, as it balances model fit and complexity, with smaller values indicating better models. According to Burnham and Anderson (2004), if the difference in AIC between two related models was greater than 10, the model with the lowest AIC was considered the best supported. However, if the difference was smaller than 10, the simpler model (with fewer parameters) was selected. Using the selected models, the effects of the covariates were evaluated by generating predictions of the response variable both overall (excluding random effects) and separately for each replicate (including random effects). This approach allows assessment of the general trend as well as replicate-specific variability.

## 3 Results

### 3.1 Mycelial growth, pycnidia development and α-conidia production at different temperatures

**Figure 1:**
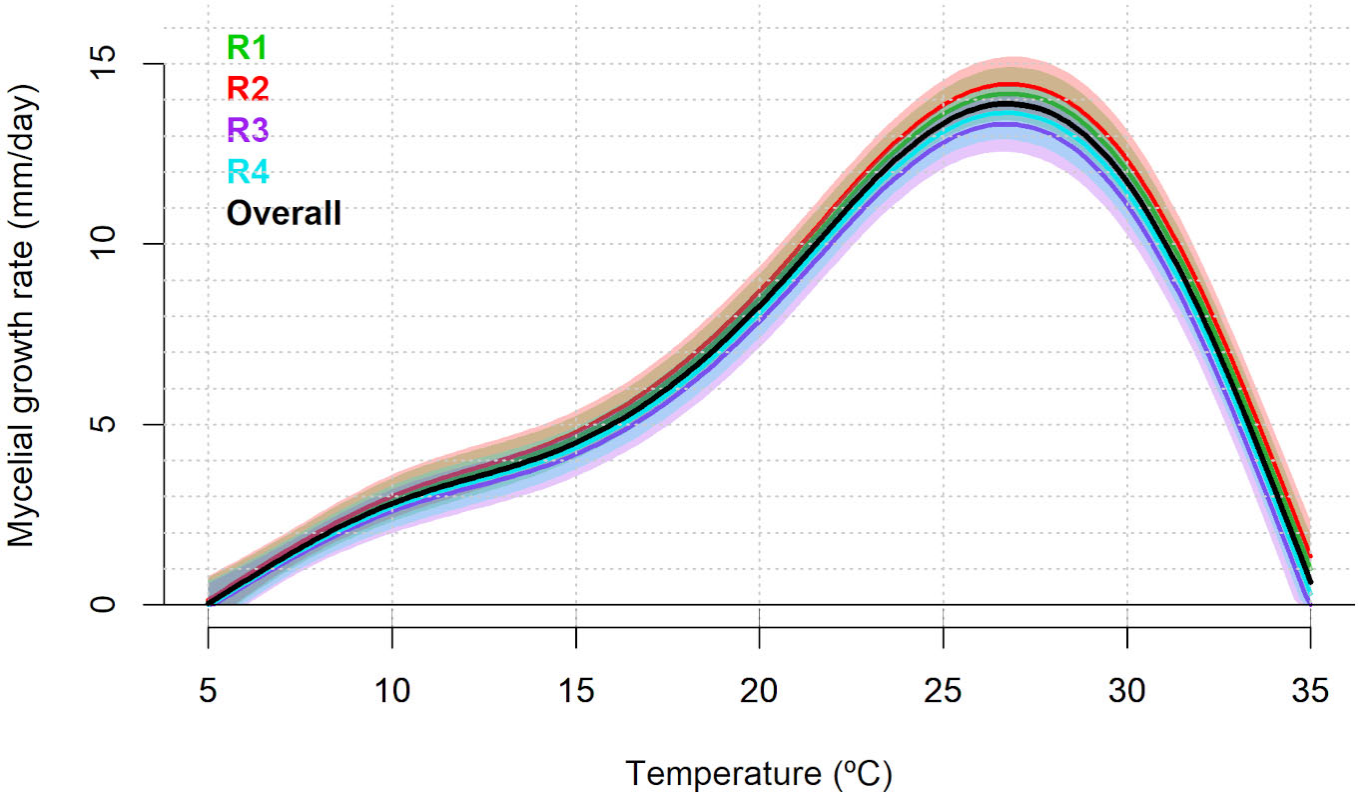
Predicted mycelial growth rate (mm day^–1^) of *Diaporthe amygdali* as a function of temperature (°C) based on the random smooth model. Overall trend (black) and individual replicate responses (colored lines) with shaded 95% confidence intervals. Replicate R1 and R2: Isolate PHAL-4. Replicate R3 and R4: isolate PHAL-45.

The observed individual values (per Petri plate) of radial mycelial growth rate (mm day^−1^), the total number of mature pycnidia, and the number of α-conidia produced per pycnidium are presented in **Supplementary Figures 1–3**, respectively. For mycelial growth data, the random smooth model had the best fit with an AIC of 195.311 and an adjusted R^2^ of 0.978 (**Supplementary Table 2**). The non-linear effect of temperature was significant (p<0.001). The random effect associated with the experimental replicates was also statistically significant (p<0.001; **Supplementary Table 3**). According to the predicted overall model trend, the mycelial growth rate progressively increased within the temperature range of 5°C to approximately 27°C. Beyond 27°C, a pronounced decrease was observed, with predicted growth values at 35°C similar to those at 5°C. The optimal mycelial growth was predicted at 26.71°C, with a maximum growth rate of 13.89 mm day^−1^. The replicate-specific predicted optimal temperatures and corresponding maximum growth rates are shown in **Table 1** and **Figure 1**.

**Table 1:** The replicate-specific predicted optimal temperatures and corresponding maximum growth rates, number of pycnidia per colony and number of α-conidia produced per pycnidium.

Mycelial growth			
Replicate	Maximum growth rate (mm day^–1^)	95% Confidence Interval (CI) (mm day^–1^)	Optimal temp. (°C)
R1	14.17	13.43 - 14.91	26.86
R2	14.44	13.68 - 15.20	26.86
R3	13.33	12.56 - 14.09	26.71
R4	13.64	12.90 - 14.38	26.71

Pycnidia development			
Replicate	Maximum number of pycnidia per colony	95% CI (number of pycnidia per colony)	Optimal temp. (°C)
R1	587	386 - 894	16.46
R2	781	514 - 1186	16.46
R3	131	81 - 211	17.01
R4	267	175 - 410	16.46

α-conidia production			
Replicate	Maximum number of α-conidia per pycnidium	95% CI (number of α-conidia per pycnidium)	Optimal temp. (°C)
R1	452,403	406,501 - 503,489	23.54
R2	361,565	312,159 - 418,790	19.02
R3	1,790,664	1,408,556 - 2,276,429	18.42
R4	763,146	664,157 - 876,889	22.49

**Figure 2:**
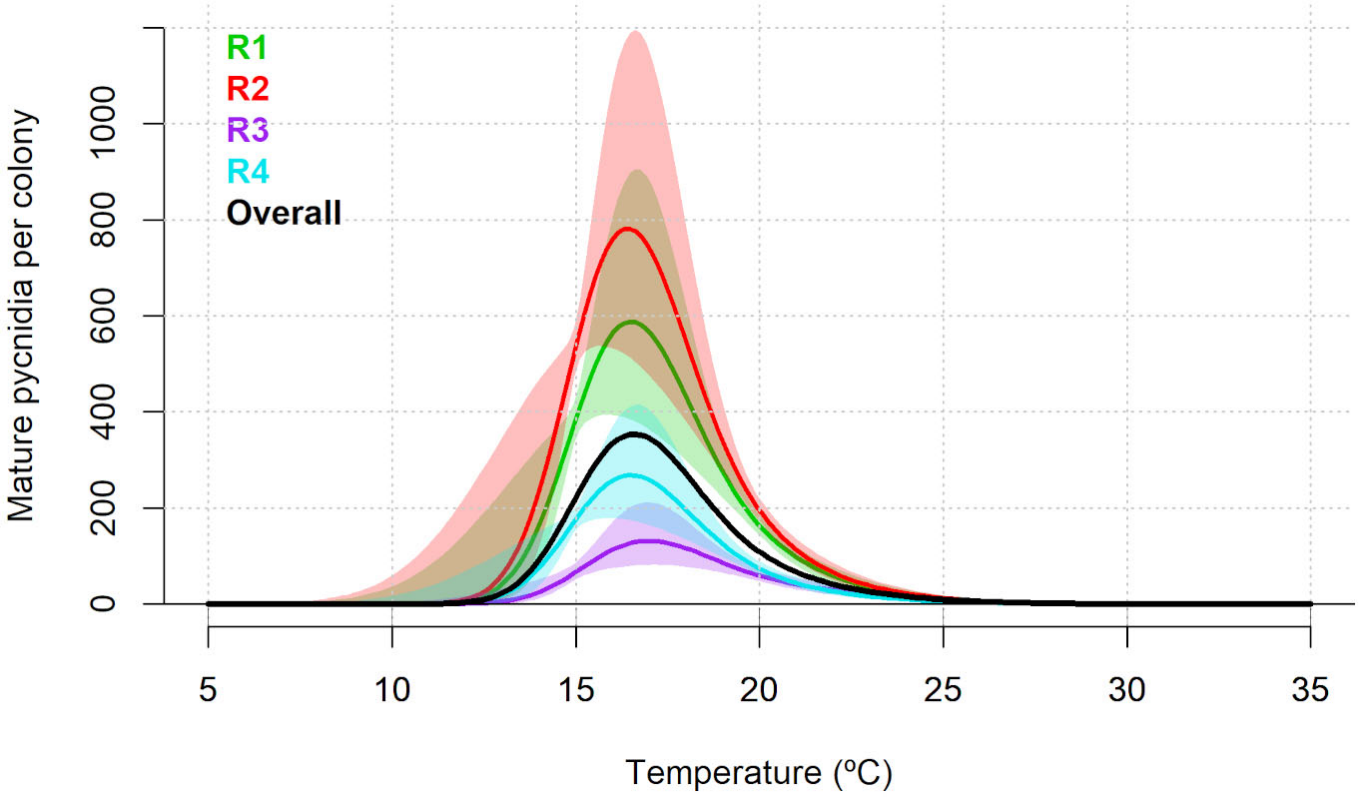
Predicted number of mature pycnidia of *Diaporthe amygdali* as a function of temperature (°C) based on the random intercept and random smooth model. Overall trend (black) and individual replicate responses (coloured lines) with shaded 95% confidence intervals. Replicate R1 and R2: Isolate PHAL-4. Replicate R3 and R4: isolate PHAL45.

For the total number of mature pycnidia per colony, the random intercept and random smooth model showed the best fit with an AIC of 1,540.614 and adjusted R^2^ of 0.800 (**Supplementary Table 2**). The non-linear effect of temperature was significant (p<0.001). Moreover, the random effect for experimental replicates included in the best-fitting model was also statistically significant (p<0.001; **Supplementary Table 3**). According to the predicted overall model trend, pycnidia production per colony was predicted to occur between 11.63 and 28.22°C, with an optimal production of 353 pycnidia per colony at 16.46°C. The replicate-specific range of temperature was from 11.33 to 28.37°C for R1, from 11.18 to 28.38°C for R2, from 12.23 to 28.67°C for R3 and from 11.63 to 27.46°C for R4. The replicate-specific predicted optimal temperatures and corresponding number of pycnidia per colony are shown in **Table 1** and **Figure 2**.

**Figure 3:**
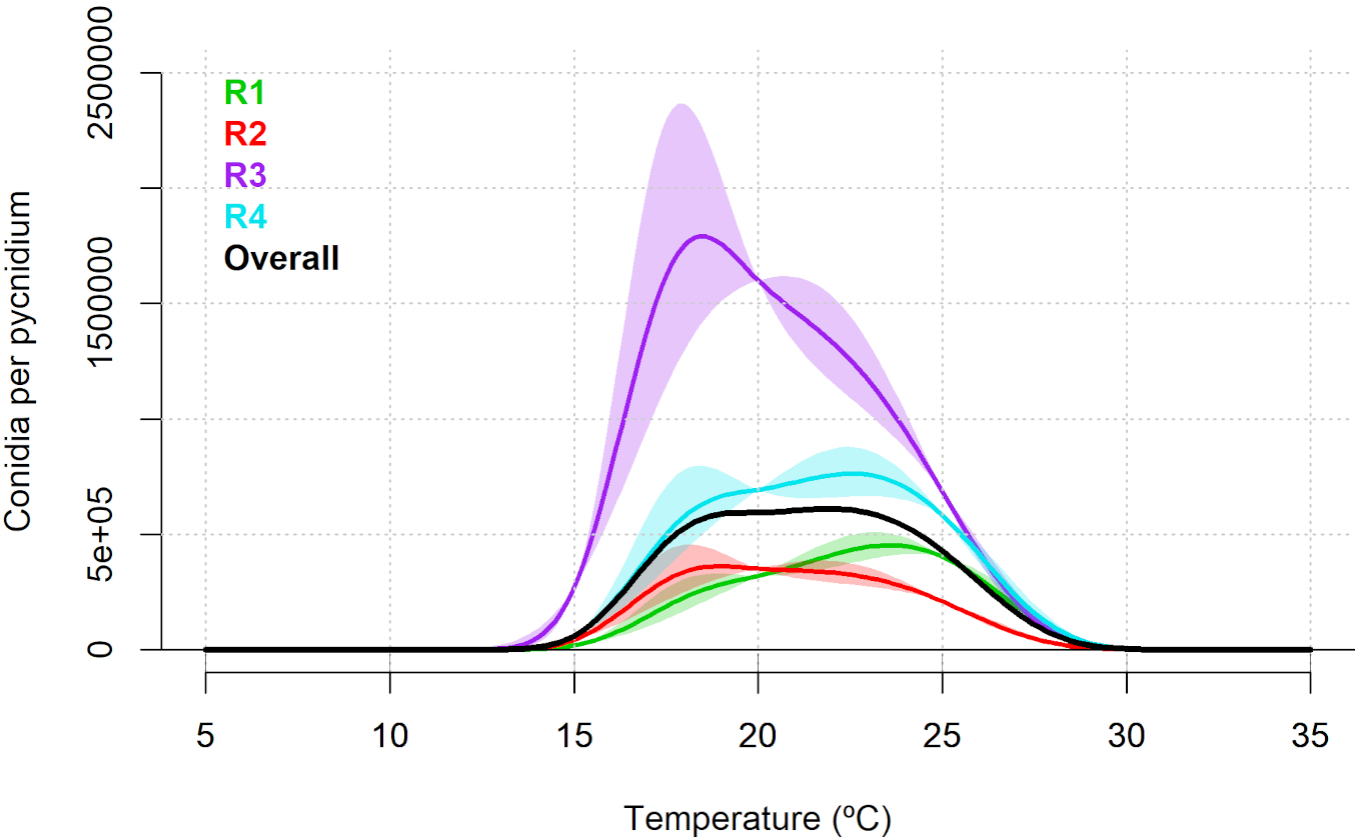
Predicted number of α-conidia of *Diaporthe amygdali* produced per pycnidium as a function of temperature (°C) based on the random intercept and random smooth model. Overall trend (black) and individual replicate responses (coloured lines) with 95% confidence intervals. Replicate R1 and R2: Isolate PHAL-4. Replicate R3 and R4: isolate PHAL45. Shaded areas represent 95% confidence intervals.

For the number of α-conidia produced per pycnidia, the random intercept and random smooth model showed the best fit with an AIC of 3,745,457 and adjusted R^2^ of 0.651 (**Supplementary Table 2**). The non-linear effect of temperature was significant (p<0.001). Moreover, the specific random effect for experimental replicates included in the selected model was also statistically significant (p<0.001; **Supplementary Table 3**). According to the predicted overall model trend, the production of α-conidia was predicted to occur between 11.21 and 32.93°C, with a maximum predicted value of 608,688 α-conidia per pycnidia at 21.55°C (**Figure 3**). The replicate-specific range of temperature was from 11.48 to 33.49°C for R1, from 11.18 to 32.89°C for R2, from 10.73 to 33.04°C for R3 and from 11.18 to 33.34°C for R4. The replicate-specific predicted optimal temperatures and corresponding number of α-conidia produced per pycnidium are shown in **Table 1** and **Figure 3**.

### 3.2 Development of mature pycnidia on almond twigs at different temperatures and days post-inoculation

**Figure 4:**
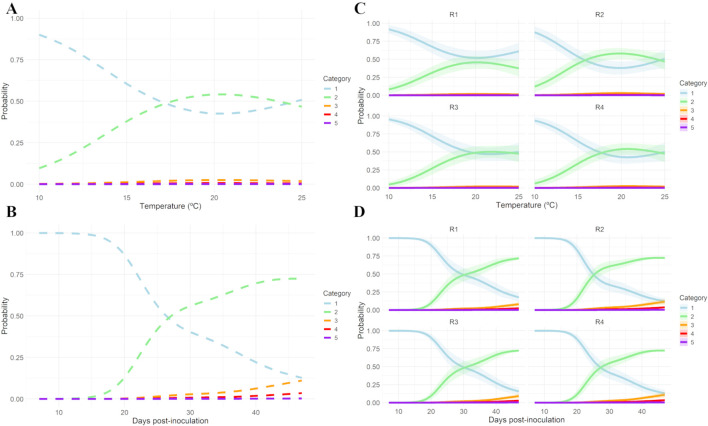
Overall **(A, B)** and individual replicate **(C, D)** predicted probabilities of mature pycnidia categories of *Diaporthe amygdali* from the random interaction effect model. **(A)** Predicted probability of occurrence of category 1 (0 mature pycnidia), 2 (1–50 mature pycnidia), 3 (51–100 mature pycnidia), 4 (101–150 mature pycnidia) and 5 (151–200 mature pycnidia) as a function of temperature (°C) for a fixed time of 24 days post-inoculation. **(B)** Predicted probability of occurrence of category 1 (0 mature pycnidia), 2 (1–50 mature pycnidia), 3 (51–100 mature pycnidia), 4 (101–150 mature pycnidia) and 5 (151–200 mature pycnidia) as a function of days post-inoculation (°C) at a constant temperature of 17.5°C. **(C)** Individual replicate predicted probabilities of mature pycnidia as a function of temperature (°C) for a fixed time of 24 days post-inoculation. **(B)** Individual replicate predicted probabilities of mature pycnidia as a function of days post-inoculation (°C) at a constant temperature of 17.5°C. Replicate R1 and R2: Isolate PHAL-4. Replicate R3 and R4: isolate PHAL45. Shaded areas represent 95% confidence intervals.

The observed probability of occurrence, calculated as the relative frequency, for each pycnidia abundance category and for each combination of temperature level (°C) and post-inoculation time (h), per each experimental replicate, is presented in **Supplementary Figures 4**, **5**. The random interaction effect model showed the best fit with an AIC of 2,625.616 and adjusted R^2^ of 0.399 (**Supplementary Table 4**). Both the joint non-linear effect of temperature and days post-inoculation as well as the random effect for the experimental replicates were statistically significant (p< 0.001; **Supplementary Table 5**). According to the predicted overall model trends (**Figure 4**), the probability of occurrence of 0 pycnidia (category 1) on the almond twig fragments showed a decreasing non-linear trend between 10 and approximately 21°C (left A) and between 1 to 50 days post-inoculation (left B). In contrast, the probability of 1 to 50 pycnidia (category 2) increased across these ranges. Probabilities for categories with more than 50 pycnidia (category 3 or more) remained low throughout, though slightly higher values were predicted at around 25°C and beyond 25 days post-inoculation. The occurrence of more than 151 pycnidia per twig fragment was not predicted under any condition. Replicate-level predictions generally followed the overall pattern, with minor variations in the transition from absence to low abundance of pycnidia (**Figure 4**).

### 3.3 Conidia germination at different temperatures and periods of incubation

**Figure 5:**
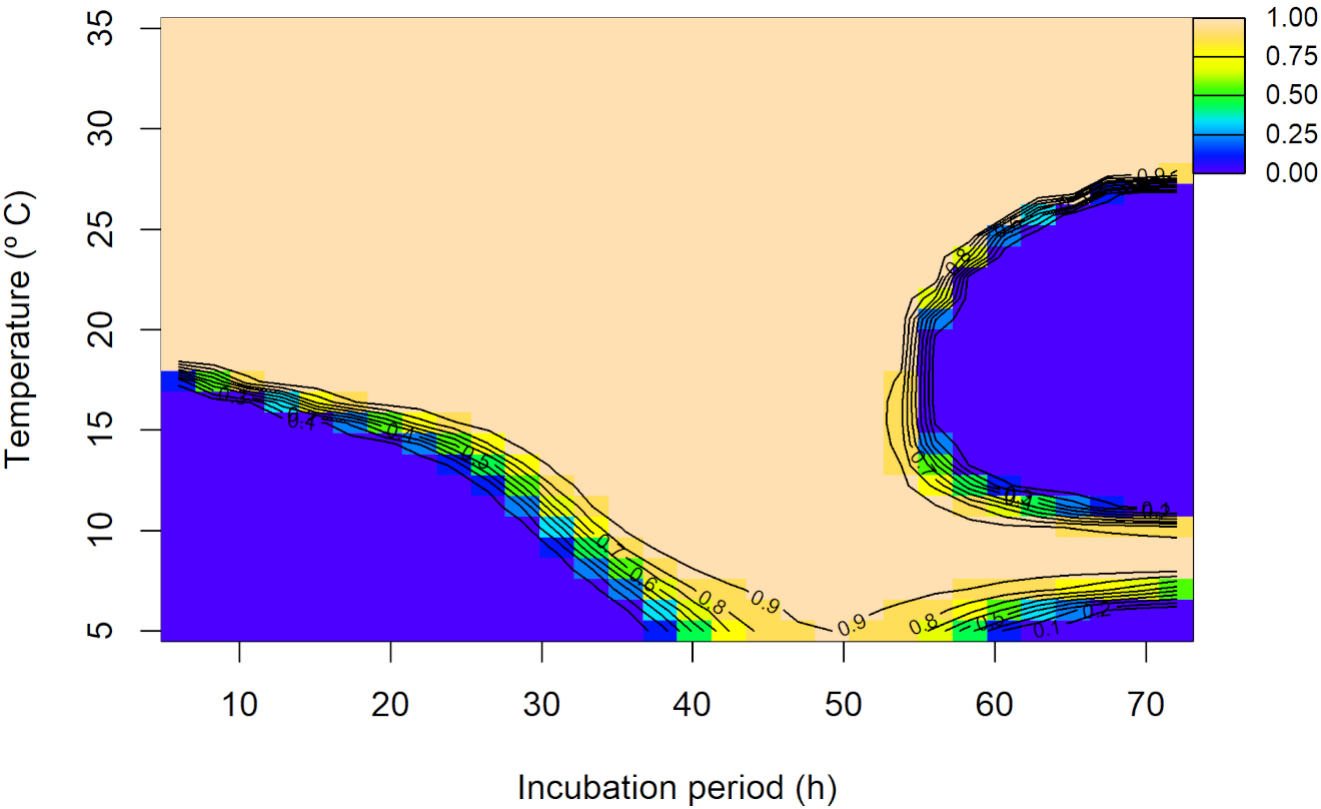
Overall predicted conidia germination (0 to 1 scale) of *Diaporthe amygdali* as a function of temperature (°C) and incubation time (h) from the random interaction effect model. Contour lines connect points with the same predicted value, with warmer colors indicating higher conidia germination and cooler colors representing lower values.

The observed individual values of the percentage of germinated conidia (%) for each evaluated temperature and incubation period, per each experimental replicate, are shown in **Supplementary Figure 6**. For conidia germination data, the random interaction model provided the best fit AIC = 8,207.99, with an adjusted R^2^ = 0.971 (**Supplementary Table 6**). The joint non-linear effect of temperature and incubation period was significant (p<0.001), highlighting their critical role in predicting conidia germination. Moreover, the random effect for the experimental replicates was also statistically significant (p<0.001; **Supplementary Table 7**). According to the overall predicted interaction trend between temperature and incubation periods, at temperatures ≤ 15°C and incubation periods ≤ 20 h, germination remained low (from 0 to 20%). At intermediate temperatures (~15 to 25°C), the trend became more complex but at high temperatures (~25 to 35°C), germination was generally high, although a decrease was predicted after 50 h of incubation. The maximum predicted germination was 100% (**Figure 5**). Replicate-level predictions closely matched the overall trend, with some deviations (**Supplementary Figure 7**).

### 3.4 Almond infection at different temperatures and wetness periods

**Figure 6:**
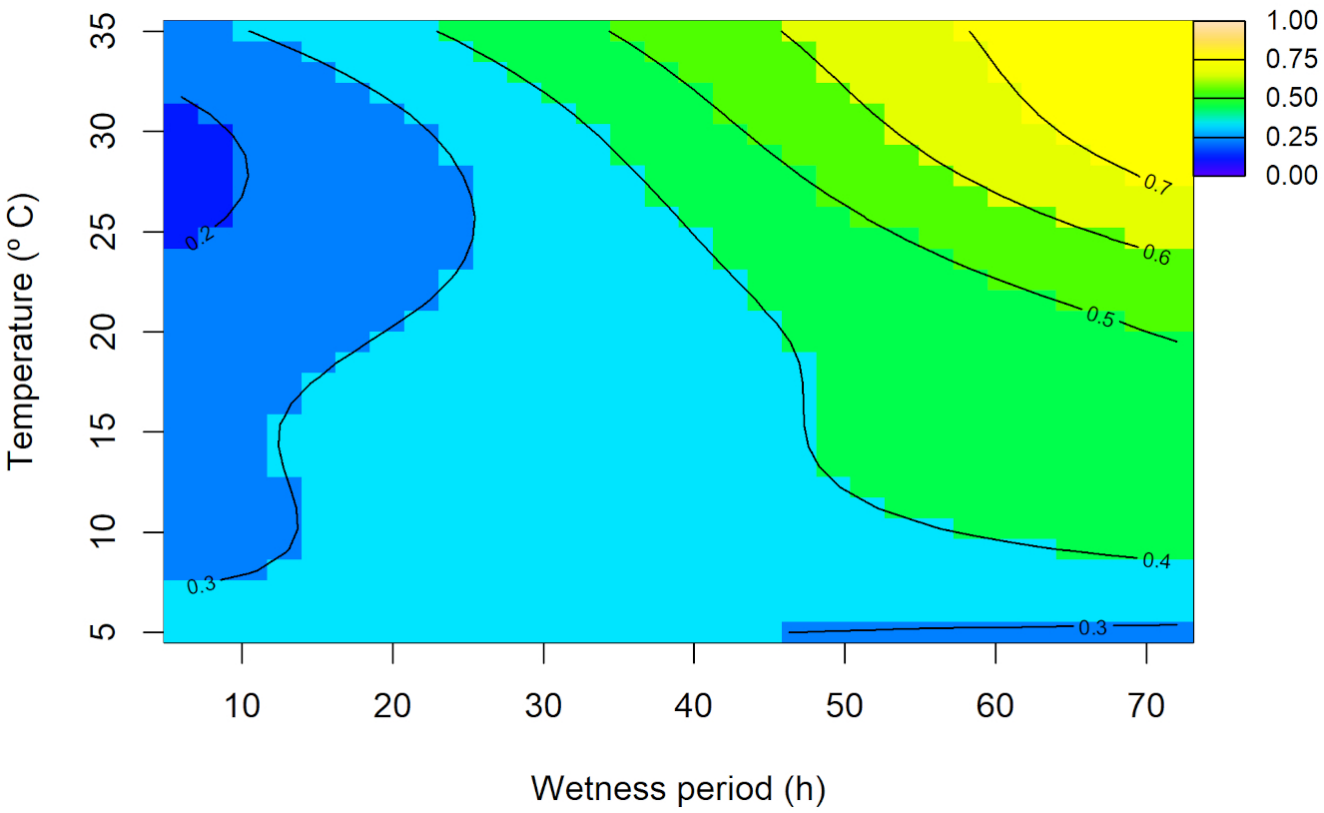
Overall predicted disease incidence on leaves (0–1 scale) of *Diaporthe amygdali* as a function of temperature (°C) and wetness period (h) from the random interaction effect model. Contour lines connect points with the same predicted value, with warmer colors indicating higher incidence and cooler colors representing lower values.

The observed values of disease incidence (%) on leaves or stems for each combination of temperature and wetness period per each experimental replicate are presented in **Supplementary Figures 8**, **9**, respectively. For disease incidence on leaves, the random interaction model provided the best fit AIC = 3,609.99, with an adjusted R^2^ = 0.443 (**Supplementary Table 8**). The joint non-linear effect of temperature and wetness period were significant (p<0.001), confirming their importance in predicting disease incidence on leaves. Moreover, the random effect for the experimental replicates was also statistically significant (p<0.001; **Supplementary Table 9**). According to the overall predicted interaction trend between temperature and wetness period (**Figure 6**), at low temperatures (~5–10°C) disease incidence on leaves remained minimal (up to 20%) independent of the wetness period. As temperature increased (up to ~25–30°C) and wetness periods extended, disease incidence on leaves progressively increased. The highest disease incidence on leaves was predicted under prolonged wetness periods and elevated temperatures (~30–35°C), where the response did not plateau but continued to increase, albeit at a reduced rate. The highest predicted disease incidence on leaves (~62%) was observed at ~33°C and 72 h of wetness period, while the lowest predicted incidence (~12%) occurred at ~28°C and 6 h. The contour lines delineated transition zones, with denser spacing observed between 30 and 60% incidence, particularly in the 25–30°C range and wetness periods beyond 50 h, indicating rapid changes in disease incidence. This pattern suggested that disease incidence on leaves is driven by a synergistic effect of temperature and wetness periods, rather than an additive relationship, underscoring the effect of their interaction. Replicate-level predictions closely matched the overall trend, with some deviations (**Supplementary Figure 10**).

**Figure 7:**
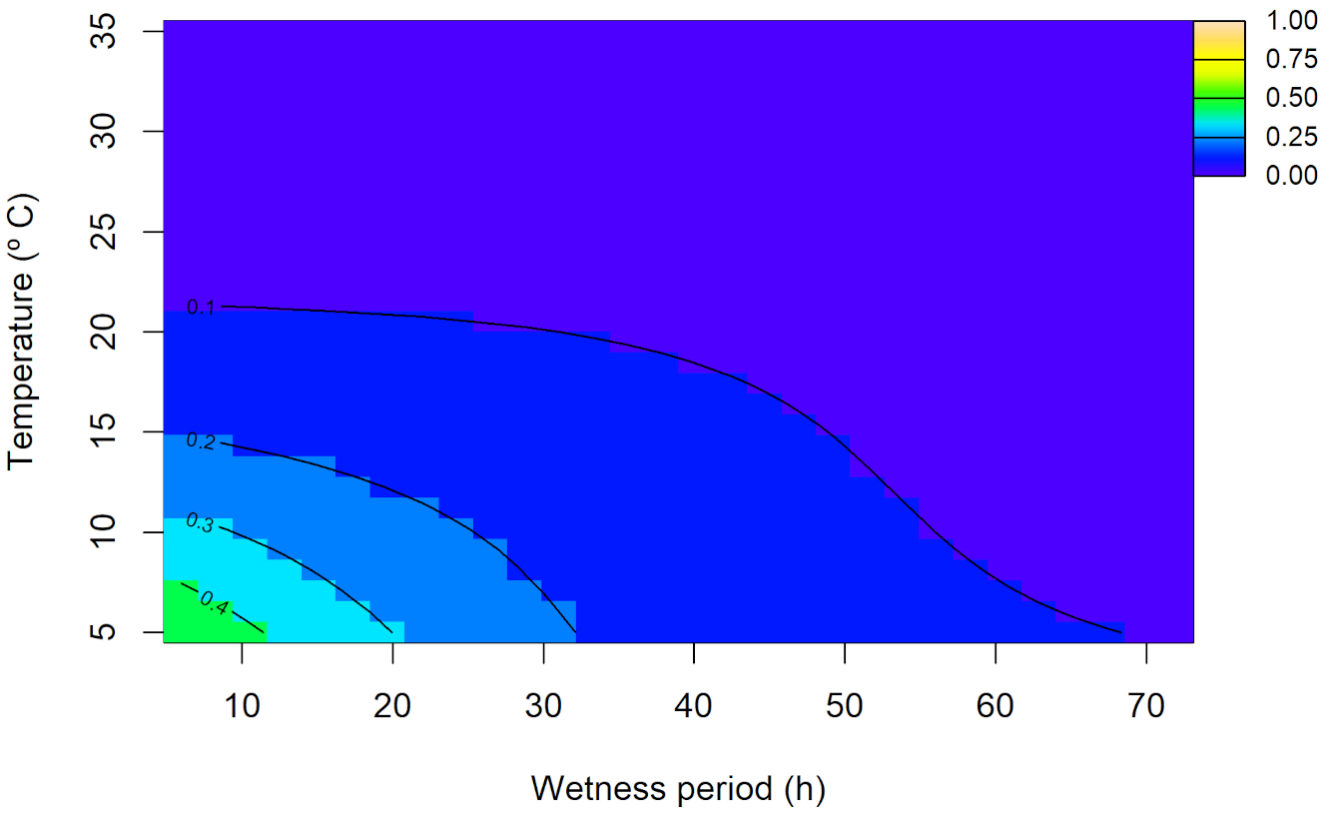
Overall predicted disease incidence on stems (0–1 scale) of *Diaporthe amygdali* as a function of temperature (°C) and wetness period (h) from the random interaction model. Contour lines connect points with the same predicted value, with warmer colors indicating higher incidence and cooler colors representing lower values.

For disease incidence on stems, the random interaction model showed the best fit AIC = 2,480.116, with a very low adjusted R^2^ = 0.191 (**Supplementary Table 8**). The joint non-linear effect of temperature and wetness periods was significant (p< 0.001), as were random effects of experimental replicates (p< 0.001; **Supplementary Table 9**). The overall predicted trend showed the highest disease incidence on stems (~47%) at low temperature (~5°C) and short wetness period (6 h), and the lowest (~3%) at high temperature (~35°C) and long wetness period (~45 h; **Figure 7**). Predictions at the replicate level deviated substantially from this overall trend (**Supplementary Figure 11**).

### 3.5 Effect of temperature on lesion development on detached almond twigs

**Figure 8:**
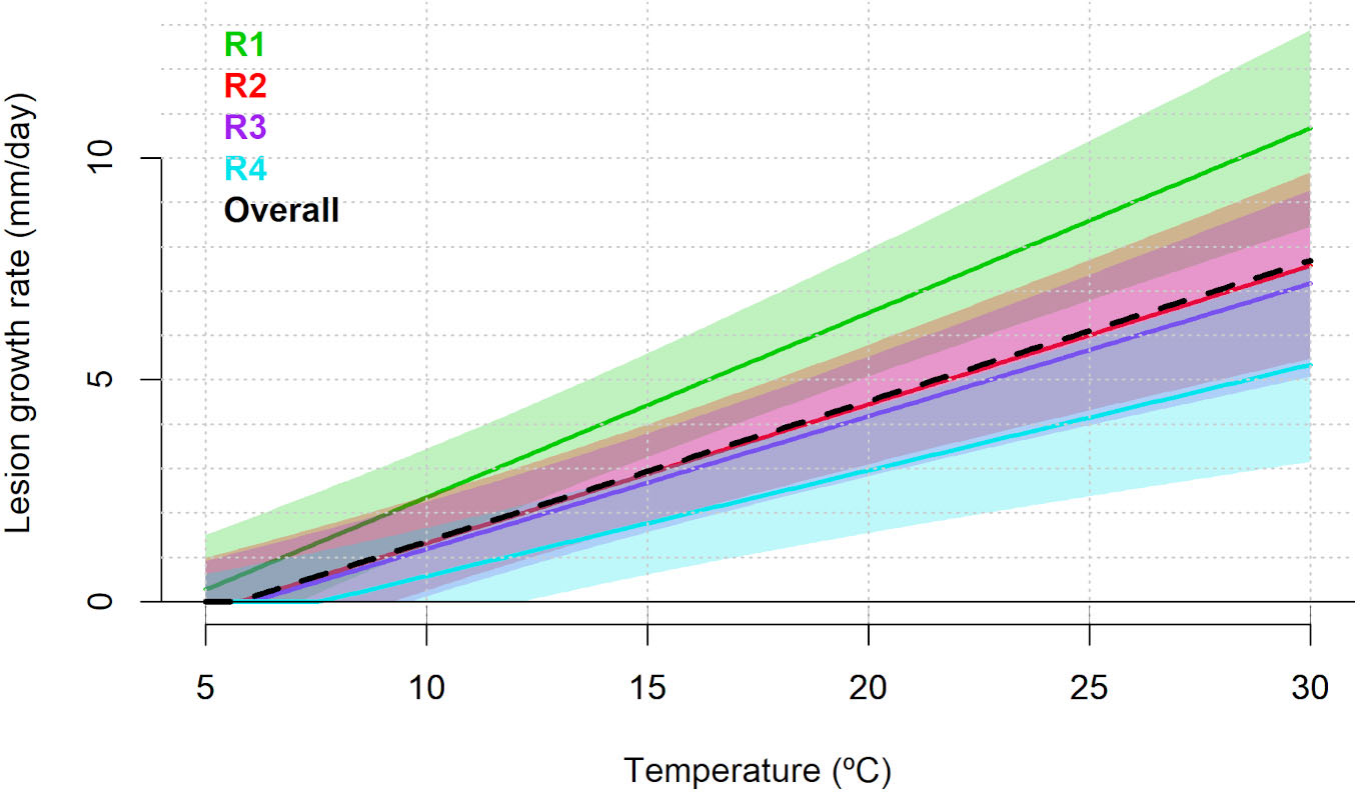
Predicted lesion growth rate (mm day^–1^) of *Diaporthe amygdali* as a function of temperature (°C) based on the random smooth model. Overall trend (black) and individual replicate responses (colored lines) with shaded 95% confidence intervals. Replicate R1 and R2: Isolate PHAL-4. Replicate R3 and R4: isolate PHAL45.

The observed individual values of lesion growth rate (mm day^-1^), for each evaluated temperature and per experimental replicate, are presented in **Supplementary Figure 12**. For lesion development data, the random smooth model provided the best fit in terms of AIC (AIC = 722.92), with an adjusted R^2^ = 0.516 (**Supplementary Table 10**). The non-linear effect of temperature was significant (p<0.001). Moreover, the random effect associated with the experimental replicates was also statistically significant (p<0.001; **Supplementary Table 11**). According to the predicted overall model trend, lesion growth rate (mm day^−1^) increased progressively across the temperature range considered in the experiment (5°C to 30°C), reaching a maximum of 7.687 mm day^−1^ at 30°C. Replicate-specific predictions also identified 30°C as the optimal temperature for maximum lesion growth rate, although the predicted peak values varied considerably among replicates: 10.667 mm day^−1^ (95% CI: 8.471 to 12.862) for R1, 7.572 mm day^−1^ (95% CI: 5.492 to 9.652) for R2, 7.169 mm day^−1^ (95% CI: 5.087 to 9.251) for R3, and 5.340 mm day^−1^ (95% CI: 3.172 to 7.508) for R4 (**Figure 8**).

## 4 Discussion

This is the first integrative study quantifying the combined effects of temperature and wetness across multiple life cycle stages of *D. amygdali* on almond, using hierarchical additive modelling.

Production of mature pycnidia on PDA occurred between 11°C to 28°C, with an optimum around 17°C. Similarly, on almond twigs, the production of mature pycnidia increased from 10°C to 21°C, with the highest probability of having more pycnidia around 20°C. These temperatures are different to those reported in previous field studies conducted on peach in USA (Lalancette and Robison, 2001), in which greater pycnidia development was observed in late summer and early autumn with temperatures around 25 and 30°C. Lalancette et al. (2003) reported cirrus production on mature pycnidia of *D. amygdali* on peach trees over a broad temperature range (1°C to 38°C), being broader than the range found in our results, but with an optimum temperature also at 17°C.

Production of α-conidia *in vitro* occurred between 11°C to 33°C, with an optimum temperature around 22°C. This result agrees with the study conducted in peach orchards by Lalancette and Robison (2001) who found the highest sporulation of *D. amygdali* in cankers at 25°C. In addition, Lalancette et al. (2003) observed a greater production of α-conidia per pycnidia of *D. amygdali* on peach at 24°C.

The germination of α-conidia was observed between 5 to 35°C and, at these temperatures, it was greater with incubation periods over 20 h, reaching the maximum germination from above 15°C and between 20 and 50 h of incubation. In agreement with our results, Zehr (1995) reported that α-conidia of *D. amygdali* germinate between 5 and 36°C with an optimum of 27 to 29°C. This wide range of temperature for α-conidia germination had been also observed in other *Diaporthe* species such as *D. eres* from hazelnut (from 10 to 40°C) (Arciuolo et al., 2021).

Lalancette et al. (2003) suggested that warmer temperatures could reduce peach leaf scar susceptibility by accelerating suberization and periderm formation. However, our findings from the leaf infection experiment indicated that warmer temperatures (30 and 35°C) with prolonged wetness periods (24–72 h) favor leaf infection. These results are consistent with Lalancette et al. (2003), who stated that, in peach, high moisture is known to inhibit protective barriers like suberin, and periderm on leaf scars, favoring *D. amygdali* infection. On the contrary, Zehr (1995) described that the infection of *D. amygdali* on peach is most common at 5 to 15°C. Regarding our results on stem infection, although the model was statistically significant, the predicted overall trend was unexpected and substantial variability among replicates further complicated epidemiological interpretation. This suggests that one-year-old almond seedling stems may not provide a reliable indicator of infection, and more lignified tissues might be needed for future studies.

Our results showed that mycelial growth of *D. amygdali* increases progressively from 5°C to 27°C, with a pronounced decrease at 35°C, and with an optimum temperature at 27°C. Gusella et al. (2023) obtained a similar temperature range for mycelial growth using almond *D. amygdali* isolates from Sicily (Italy), with an optimum around 25°C. A study of peach *D. amygdali* isolates from Brazil showed the same range of temperatures for mycelial growth, with the optimum between 20°C and 30°C (Corrêa, 2014), while López-Moral et al. (2020a) reported an optimum growth temperature of *D. amygdali* from English walnut at 26.3°C.

Finally, lesion development on detached shoots was observed between 5°C and 30°C, being the largest lesions observed around 30°C. These results are consistent with the results of Lalancette and Robison (2001) in which the greatest development of *D. amygdali* lesions in peach was observed around 30°C. Our findings are in contrast with observations of Gusella et al. (2023) about *D. amygdali* lesion development also using detached almond shoots of the cv. Soleta, where lesions developed from 5 to 35°C, with an optimum at 20.8°C. The differences observed between both studies could be due to the use of different cultivars, Soleta and Vairo, although Beluzán et al. (2022) demonstrated that both cultivars can be considered as very susceptible to *D. amygdali*.

The results of all our experiments contribute to a better understanding of how environmental factors influence the biology of *D. amygdali* in almond and, therefore, determine the cycle of twig canker and shoot blight disease in Mediterranean conditions. Temperature range for α-conidia germination and almond infection are broad and overlap (between 5 and 35°C), potentially allowing infections to occur year-round. Nevertheless, the highest infection of almond plants was observed after 72 h of wetness period, while the lowest occurred after 6 h. This could explain why *D. amygdali* is a prevalent disease in spring and autumn when rain events are more frequent, thus being also favored in coastal areas where wetness is higher than in inland almond-growing areas. Once the infection is produced, mycelial growth and lesion development are promoted by warm temperatures. But the development of cankers in field conditions is probably lower in autumn than in spring due to the loss of moisture from the bark tissues as indicated by Tuset (2000). The production of mature pycnidia on cankers has lower and narrower temperature requirements, thus suggesting an adaptation to late winter and early spring conditions. Moreover, the optimum temperature for α-conidia production in pycnidia is around 22°C.

All this information, together with the findings of Beluzán et al. (2025) about the effect of weather variables on *D. amygdali* inoculum dispersal in almond orchards, provide the basis for developing a mechanistic model to predict infection risk. Similarly, a mechanistic model to predict the infection risk of *Diaporthe ampelina*, the causal agent of Phomopsis cane and leaf spot of grapevine has been previously developed and validated using information about the inoculum dispersal and the infection conditions (Gonzalez-Dominguez et al., 2022). Mechanistic models in plant pathology require robust datasets and a detailed understanding of disease processes to described biological responses as functions of environmental variables, such as those generated in the present study. These models are a key component of decision-support systems for plant disease management, improving the timeliness and effectiveness of control strategies while reducing both economic costs and environmental impacts (Rossi et al., 2019; Lázaro et al., 2021; González-Domínguez et al., 2023). Ultimately, these models will enable informed strategies for the management of almond twig canker and shoot blight caused by *D. amygdali*.

## Data Availability

The raw data supporting the conclusions of this article will be made available by the authors, without undue reservation.
