# Supplementary material for: Biology and epidemiology of Diaporthe amygdali: understanding how environmental factors influence fungal growth, sporulation, infection and lesion development on almond
Source: Front Plant Sci. 2025 Dec 10;16:1717223. doi: 10.3389/fpls.2025.1717223 (PMC12727959; doi:10.3389/fpls.2025.1717223)
Supplement: Supplementary file 1 [file DataSheet1.docx]

Supplementary Material

# Supplementary Figures and Tables

**Supplementary 1.** Model specification for each experiment and response variable in terms of its distribution, link function, covariate structure, and random effects

| Experiment | Response variable | Distribution | Link fuction | Covariate structure | Random effects structure |
| --- | --- | --- | --- | --- | --- |
| Mycelial growth, pycnidia development and α conidia production at different temperatures (Temp). | Mycelial growth rate (mm day⁻¹) | Gaussian | Identity | f(Temp) | r(Replicate) |
|  | Number mature pycnidia | Poisson | Logarithm |  | r(Temp,Replicate) |
|  | Number conidia | Poisson | Logarithm |  | r(Replicate) + r(Temp,Replicate) |
| Development of mature pycnidia on almond twigs at different temperatures (Temp) and days post-inoculation (Inoc) | Mature pycnidia abundance (0= no mature pycnidia; 1= 1 to 50 mature pycnidia; 2= 51 to 100 mature pycnidia; 3= 101 to 150 matura pycnida; 4=151 to 200 mature pycnidia) | Ordinal categorical | Identity | f(Temp,Inoc) | r(Replicate) |
|  |  |  |  |  | r(Temp,Replicate) + r(Inoc,Replicate) |
|  |  |  |  |  | r(Replicate) + r(Temp,Replicate) + r(Inoc,Replicate) |
|  |  |  |  |  | r(Temp, Inoc, Replicate) |
| Conidia germination at different temperatures (Temp) and periods of incubation (Incub). | Conidia germination (%) | Binomial | Logit | f(Temp,Incub) | r(Replicate) |
|  |  |  |  |  | r(Temp,Replicate) + r(Incub,Replicate) |
|  |  |  |  |  | r(Replicate)+ r(Temp,Replicate) + r(Incub,Replicate) |
|  |  |  |  |  | r(Temp, Incub, Replicate) |
| Almond infection at different temperatures (Temp) and wetness periods (Wetness). | Disease incidence on leaves (%) | Binomial | Logit | f(Temp,Wetness) | r(Replicate) |
|  |  |  |  |  | r(Temp,Replicate) + r(Inoc,Replicate) |
|  | Disease incidence on stems (%) |  |  |  | r(Replicate) + r(Temp,Replicate) + r(Wetness,Replicate) |
|  |  |  |  |  | r(Temp, Wetness, Replicate) |
| Lesion development on detached almond twigs at different temperatures (Temp). | Lesion growth rate (mm day⁻¹) | Gaussian | Identity | f(Temp) | r(Replicate) |
|  |  |  |  |  | r(Temp,Replicate) |
|  |  |  |  |  | r(Replicate) + r(Temp,Replicate) |


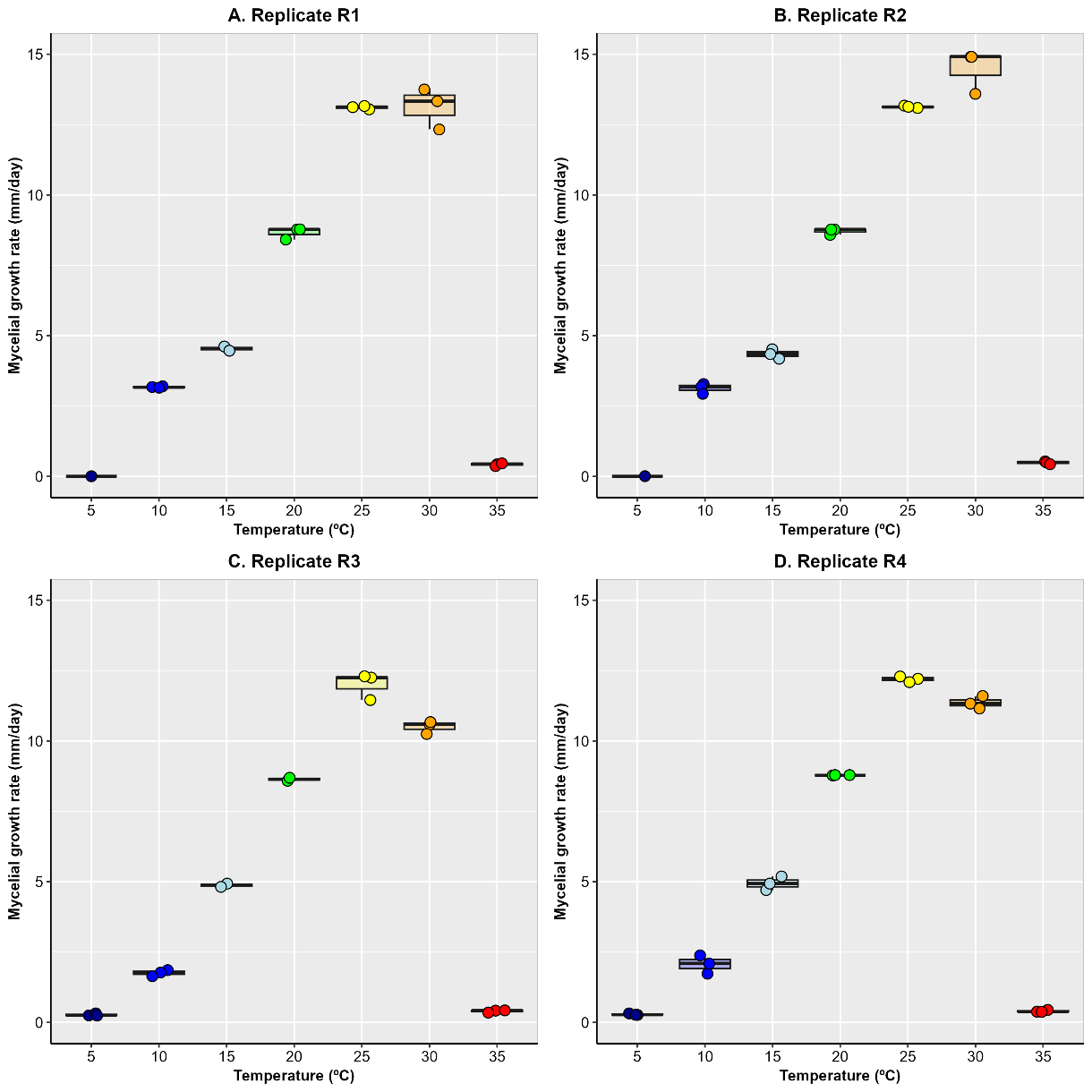


**Supplementary 2.** Exploratory analysis of mycelial growth rate (mm day-1) at different temperatures (5, 10, 15, 20, 25, 30, and 35 ºC) per replicate (replicate R1 and R2: isolate PHAL-4, replicate R3 and R4: isolate PHAL-45).


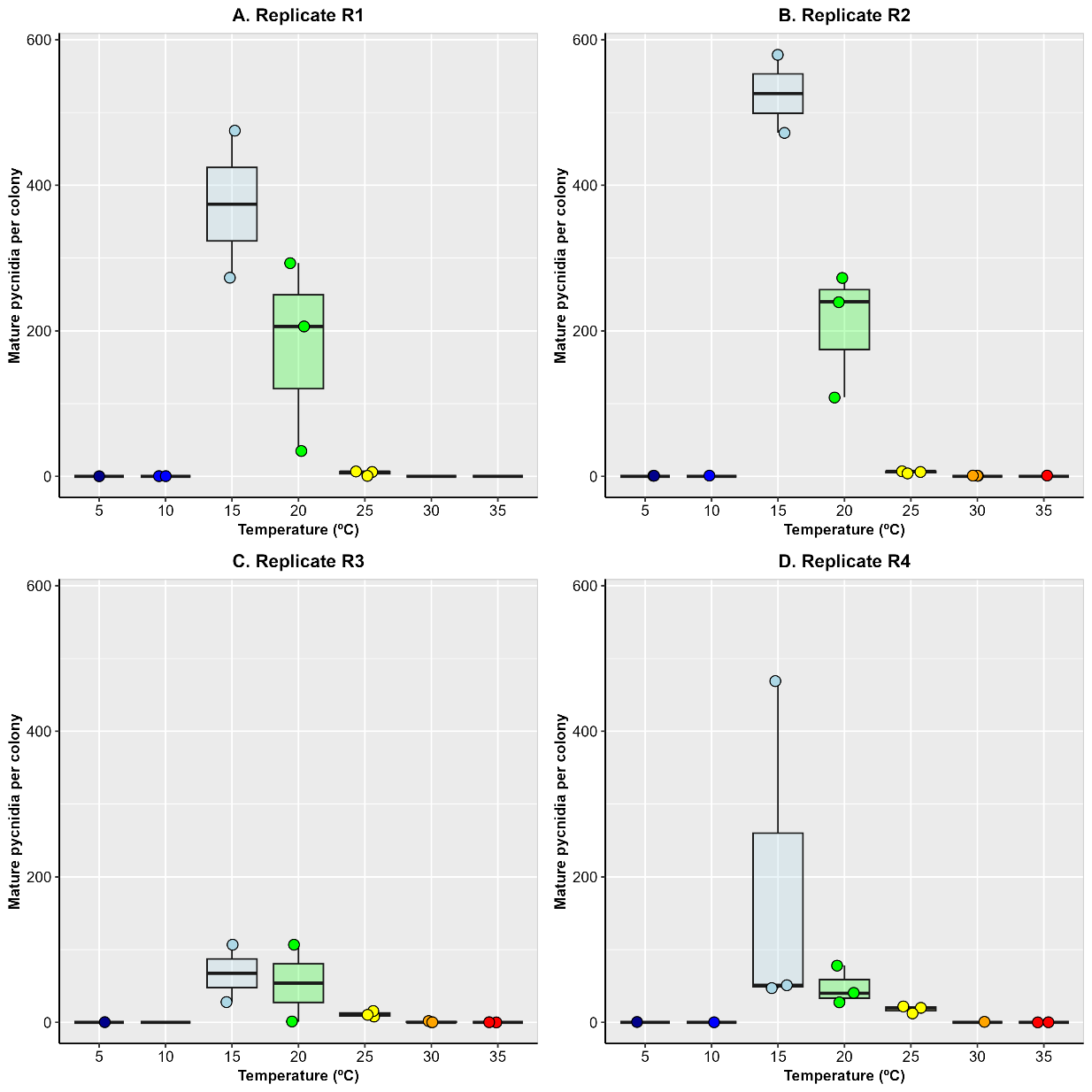


**Supplementary 3.** Exploratory analysis of the total number of mature pycnidia at different temperatures (5, 10, 15, 20, 25, 30, and 35 ºC) per replicate (replicate R1 and R2: isolate PHAL-4, replicate R3 and R4: isolate PHAL-45).


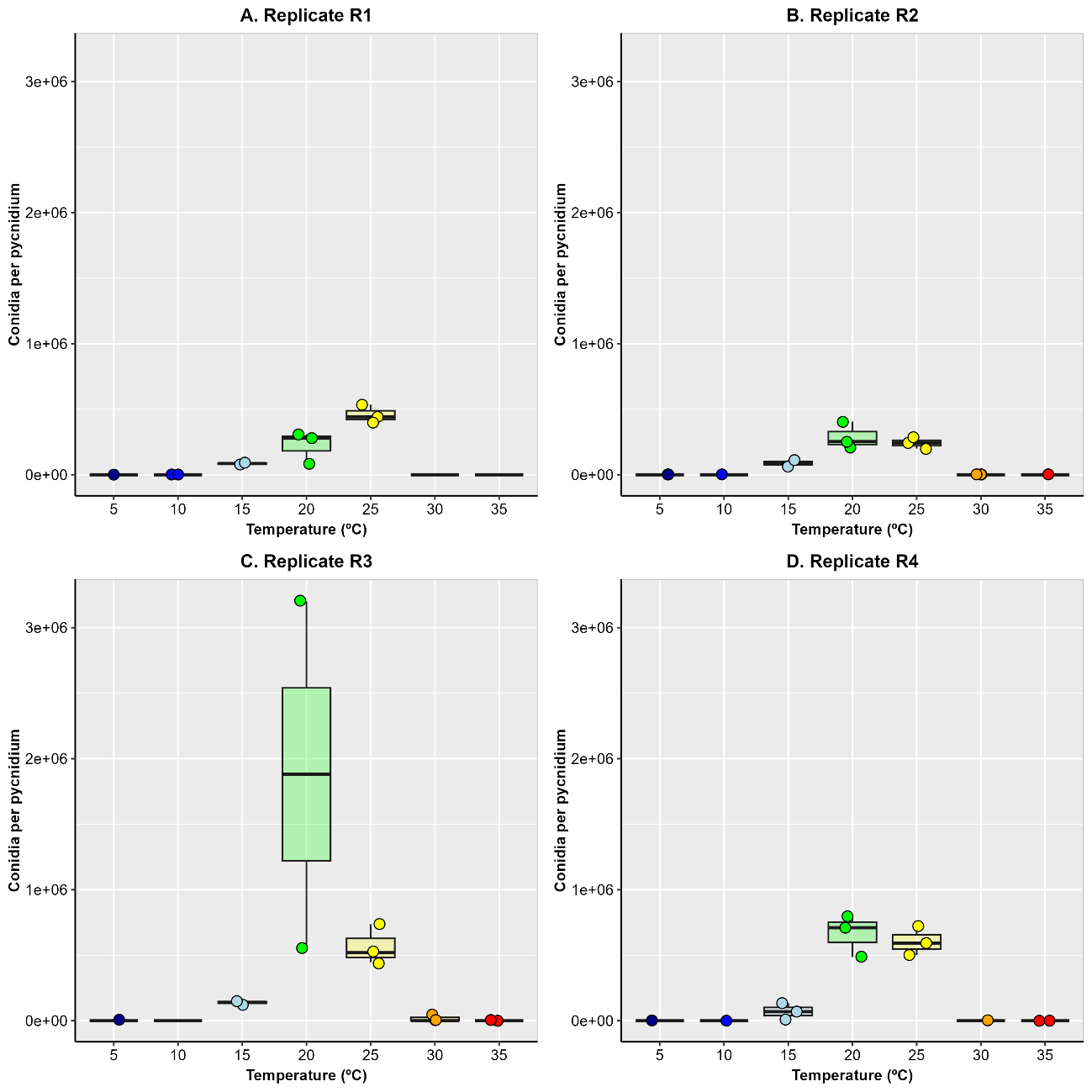


**Supplementary 4.** Exploratory analysis of number of α-conidia produced per pycnidium at different temperatures (5, 10, 15, 20, 25, 30, and 35 ºC) per replicate (replicate R1 and R2: isolate PHAL-4, replicate R3 and R4: isolate PHAL-45).

**Supplementary 5.** Comparison and performance in terms of Akaike Information Criterion (AIC), adjusted R², deviance, and the number of parameters of the three statistical models for mycelial growth rate (mm day⁻¹), total number of mature pycnidia and the number of α-conidia produced per pycnidium.

| Mycelial growth rate | | | | | |
| --- | --- | --- | --- | --- | --- |
| Model specification | AIC | | R2_adjusted | Deviance | Num_parameters |
| Mycelial growth rate∼  f(Temp)^[[1]](#footnote-1)^ +  r(Replicate)^[[2]](#footnote-2)^ | 202.137 | | 0.976 | 0.978 | 9 |
| Mycelia growth rate∼  f(Temp)+  r(Temp,Replicate)^[[3]](#footnote-3)^ | **195.311** | | **0.978** | **0.980** | **9** |
| Mycelial growth rate∼  f(Temp)+  r(Replicate)+  r(Temp,Replicate) | 195.313 | | 0.978 | 0.980 | 13 |
| Number mature pycnidia per colony | | | | | |
| Model specification | | AIC | R2_adjusted | Deviance | Num_parameters |
| Number mature pycnidia∼  f(Temp) +  r(Replicate) | | 1,616.386 | 0.805 | 0.894 | 9 |
| Number mature pycnidia ∼  f(Temp)+  r(Temp,Replicate) | | 1,719.145 | 0.797 | 0.887 | 9 |
| Number mature pycnidia ∼  f(Temp)+  r(Replicate)+  r(Temp,Replicate) | | **1,540.614** | **0.800** | **0.900** | **13** |
| Number of α conidia produced per pycnidium | | | | | |
| Model specification | | AIC | R2_adjusted | Deviance | Num_parameters |
| Number conidia∼  f(Temp) +  r(Replicate) | | 4,506,107 | 0.596 | 0.881 | 9 |
| Number conidia∼  f(Temp)+  r(Temp,Replicate) | | 4,897,371 | 0.562 | 0.871 | 9 |
| Number conidia∼  f(Temp)+  r(Replicate)+  r(Temp,Replicate) | | **3,745,457** | **0.651** | **0.901** | **13** |

*Best fit models are in bold based on the lowest AIC value.

**Supplementary 6.** Summary of the best model results for mycelial growth rate (mm day⁻¹), for the number of mature pycnidia and for the number of α conidia produced per pycnidia

| Mycelial growth rate | | | | | | | | |
| --- | --- | --- | --- | --- | --- | --- | --- | --- |
| Category | Term | Estimate | Std_Error | t_value | EDF | Ref_df | F | P_value |
| Parametric Coefficients | (Intercept) | 5.91 | 0.219 | 27.033 | - | - | - | 0*** |
| Smooth Terms | f(Temp) | - | - | - | 3.993 | 4 | 783.287 | 0*** |
| Smooth Terms | r(Temp,Replicate) | - | - | - | 2.631 | 3 | 7.092 | 0*** |
| Model Summary | R-squared (adjusted) | 0.978 | - | - | - | - | - | - |
| Model Summary | Deviance explained | 0.98 | - | - | - | - | - | - |
| Model Summary | -REML | 108.124 | - | - | - | - | - | - |
| Model Summary | Scale estimation | 0.578 | - | - | - | - | - | - |
| Model Summary | Sample size | 81 | - | - | - | - | - | - |
| Number of mature pycnidia per colony | | | | | | | | |
| Category | Term | Estimate | Std_Error | t_value | EDF | Ref_df | F | P_value |
| Parametric Coefficients | (Intercept) | -5.153 | 2.088 | -2.468 | - | - | - | 0.014** |
| Smooth Terms | f(Temp) | - | - | - | 3.899 | 3.991 | 326.932 | 0*** |
| Smooth Terms | r(Replicate) | - | - | - | 2.964 | 3 | 1870652.684 | 0*** |
| Smooth Terms | r(Temp,Replicate) | - | - | - | 2.924 | 3 | 876146.735 | 0*** |
| Model Summary | R-squared (adjusted) | 0.8 | - | - | - | - | - | - |
| Model Summary | Deviance explained | 0.9 | - | - | - | - | - | - |
| Model Summary | -REML | 794.45 | - | - | - | - | - | - |
| Model Summary | Scale estimation | 1 | - | - | - | - | - | - |
| Model Summary | Sample size | 80 | - | - | - | - | - | - |
| Number of α conidia produced per pycnidia | | | | | | | | |
| Category | Term | Estimate | Std_Error | Z_value | EDF | Ref_df | Chi_sq | P_value |
| Parametric Coefficients | (Intercept) | -1.197 | 2.232 | -0.536 | - | - | - | 0.592 |
| Smooth Terms | f(Temp) | - | - | - | 3.926 | 3.995 | 4745664.104 | 0*** |
| Smooth Terms | r(Replicate) | - | - | - | 3 | 3 | 6500330144.962 | 0*** |
| Smooth Terms | r(Temp,Replicate) | - | - | - | 3 | 3 | 50542403475.805 | 0*** |
| Model Summary | R-squared (adjusted) | 0.651 | - | - | - | - | - | - |
| Model Summary | Deviance explained | 0.901 | - | - | - | - | - | - |
| Model Summary | -REML | 1872796.88 | - | - | - | - | - | - |
| Model Summary | Scale estimation | 1 | - | - | - | - | - | - |
| Model Summary | Sample size | 80 | - | - | - | - | - | - |

Estimate is the effect size; Std_Error is the uncertainty around the estimate; t_value/Z_value indicates how far the estimate is from zero; EDF represents the flexibility of smooth terms; Ref_df is the reference degrees of freedom; F/Chi_sq tests the significance of terms; P_value indicates statistical significance, with values marked as follows: *** for p ≤ 0.001, ** for p ≤ 0.01, * for p ≤ 0.05, . for p ≤ 0.1, and no symbol for p > 0.1.


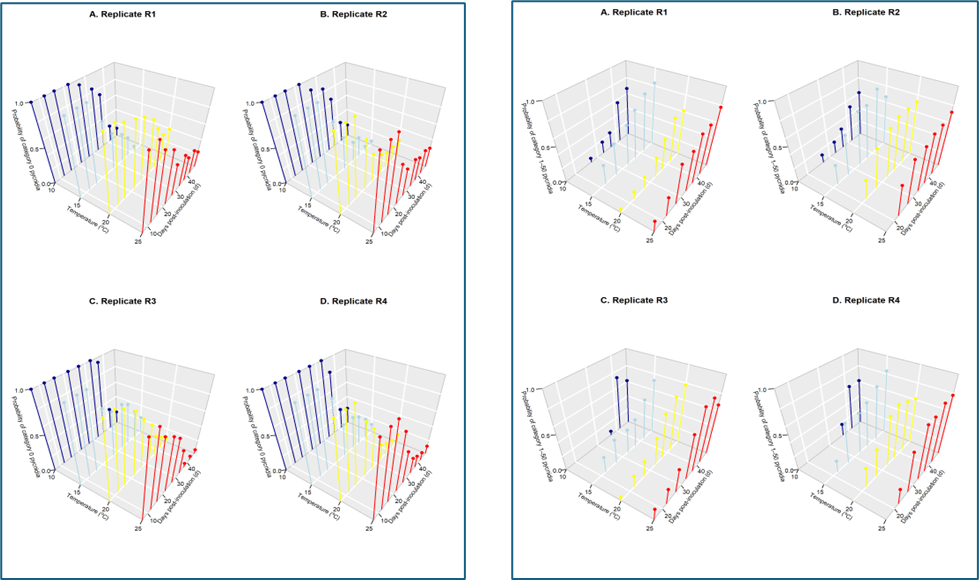


**Supplementary 7.** Exploratory analysis of the probability of occurrence of 0 (left) and 1 to 50 (right) mature pycnidia, calculated as the relative frequency within each abundance category, for each combination of temperature (10, 15, 20 and 25 ºC) and days post-inoculation (7, 12, 17, 22, 27, 32, 37,42 and 47 days) per replicate (replicate R1 and R2: isolate PHAL-4, replicate R3 and R4: isolate PHAL-45).


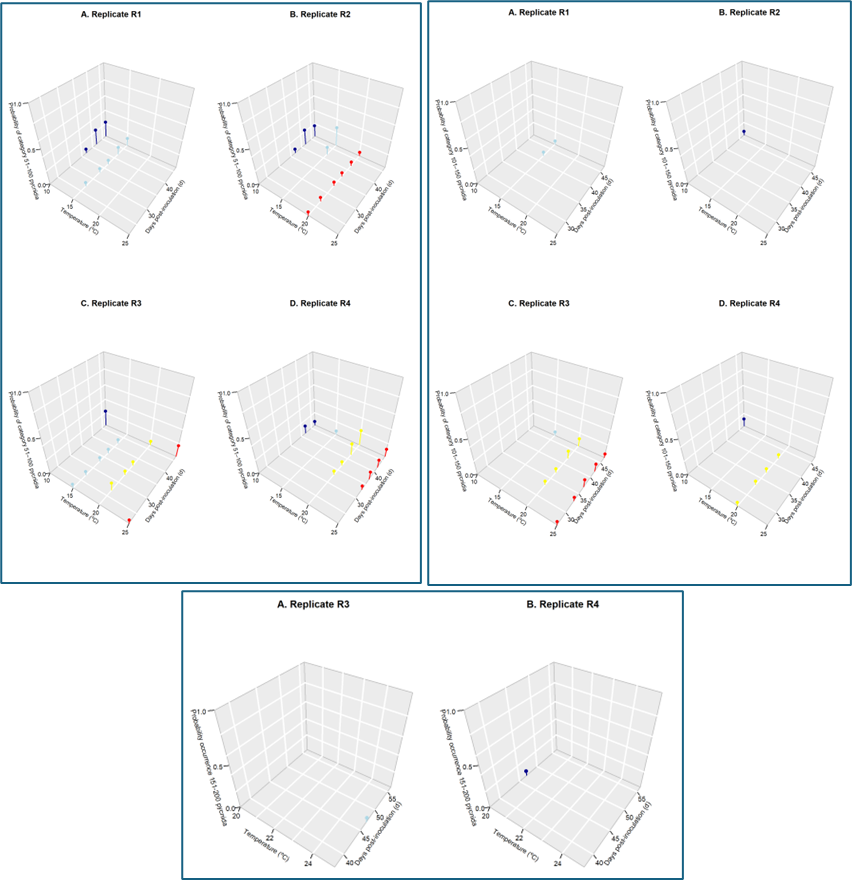


**Supplementary 8.** Exploratory analysis of the probability of occurrence of 51 to 100 (left), 101 to 150 (right) and 151-200 (down) mature pycnidia, calculated as the relative frequency within each abundance category, for each combination of temperature (10, 15, 20 and 25) and days post-inoculation (7, 12, 17, 22, 27, 32, 37,42 and 47 days) per replicate (replicate R1 and R2: isolate PHAL-4, replicate R3 and R4: isolate PHAL-45).

**Supplementary 9.** Comparison and performance in terms of Akaike Information Criterion (AIC), deviance, and the number of parameters of the four statistical models for the probability of occurrence of mature pycnidia

| Model specification | AIC | Deviance | Num_parameters |
| --- | --- | --- | --- |
| Mature pycnidia∼  f(Temp,Days)^[[4]](#footnote-4)^+  r(Replicate)^[[5]](#footnote-5)^ | 2,669.615 | 0.380 | 25 |
| Mature pycnidia∼  f(Temp,Days)+  r(Temp,Replicate) ^[[6]](#footnote-6)^+  r(Days,Replicate)^[[7]](#footnote-7)^ | 2,665.794 | 0.381 | 29 |
| Mature pycnidia∼  f(Temp,Days)+  r(Replicate)+  r(Temp,Replicate) +  r(Days,Replicate) | 2,636.782 | 0.393 | 33 |
| Mature pycnidia∼  f(Temp,Days)+  r(Temp,Days,Replicate)^[[8]](#footnote-8)^ | **2,625.616** | **0.399** | **84** |

^*^Best fit model is in bold based on the lowest AIC value.

**Supplementary 10.** Summary of the random interaction model for probability of occurrence of mature pycnidia

| Category | Term | Estimate | Std_Error | Z_value | EDF | Ref_df | Chi_sq | P_value |
| --- | --- | --- | --- | --- | --- | --- | --- | --- |
| Parametric Coefficients | (Intercept) | -2.853 | 0.329 | -8.674 | - | - | - | 0*** |
| Smooth Terms | f(Temp,Days) | - | - | - | 13.486 | 15.518 | 357.589 | 0*** |
| Smooth Terms | r(Temp,Days,Rep) | - | - | - | 10.81 | 63 | 65.749 | 0*** |
| Model Summary | Deviance explained | 0.399 | - | - | - | - | - | - |
| Model Summary | -REML | 1317.771 | - | - | - | - | - | - |
| Model Summary | Scale estimation | 1 | - | - | - | - | - | - |
| Model Summary | Sample size | 2592 | - | - | - | - | - | - |

Estimate is the effect size; Std_Error is the uncertainty around the estimate; Z_value indicates how far the estimate is from zero; EDF represents the flexibility of smooth terms; Ref_df is the reference degrees of freedom; Chi_sq tests the significance of terms; P_value indicates statistical significance, with values marked as follows: *** for p ≤ 0.001, ** for p ≤ 0.01, * for p ≤ 0.05, . for p ≤ 0.1, and no symbol for p > 0.1.


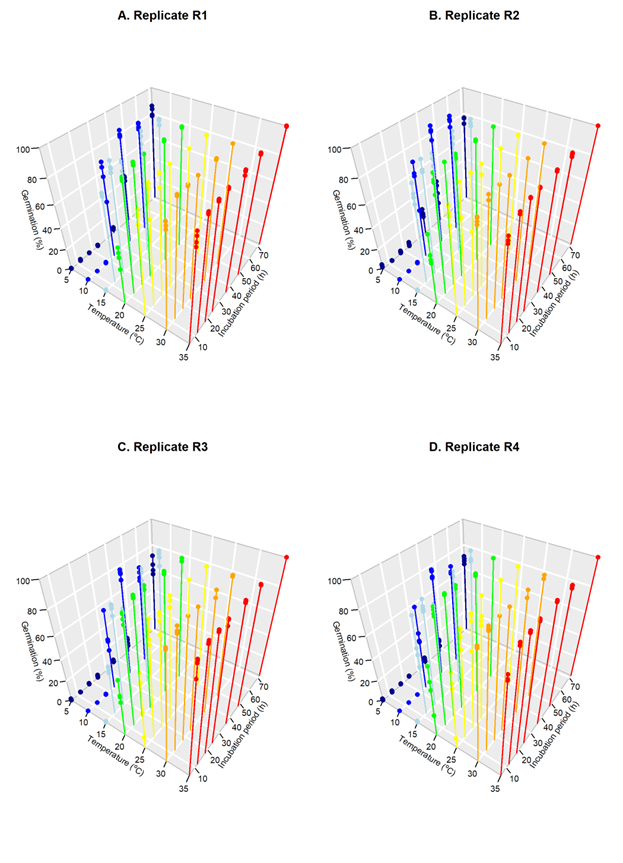


**Supplementary 11.** Exploratory analysis of conidia germination (%) for each combination of temperature (5, 10, 15, 20, 25, 30, and 35 ºC) and incubation period (6, 12, 18, 24, 36 and 48 h) per replicate (replicate R1 and R2: isolate PHAL-4, replicate R3 and R4: isolate PHAL-45).

**Supplementary 12.** Comparison and performance in terms of Akaike Information Criterion (AIC), adjusted R², deviance, and the number of parameters of the four statistical models for conidia germination (%)

| Model specification | AIC | R2_adjusted | Deviance | Num_parameters |
| --- | --- | --- | --- | --- |
| Conidia Germination∼  f(Temp,Incub)^[[9]](#footnote-9)^ +  r(Replicate)^[[10]](#footnote-10)^ | 9,177.422 | 0.967 | 0.939 | 29 |
| Conidia Germination∼  f(Temp,Incub)+  r(Temp,Replicate)^[[11]](#footnote-11)^+  r(Incub,Replicate)^[[12]](#footnote-12)^ | 9,239.242 | 0.966 | 0.939 | 33 |
| Conidia Germination∼  f(Temp,Incub)+  r(Replicate)+  r(Temp,Replicate) +  r(Incub,Replicate) | 9,063.016 | 0.967 | 0.940 | 37 |
| Conidia Germination∼  f(Temp,Incub)+  r(Temp,Incub,Replicate)^[[13]](#footnote-13)^ | **8,207.996** | **0.971** | **0.948** | **100** |

^*^Best fit model is in bold based on the lowest AIC value.

**Supplementary 13.** Summary of the random interaction model for conidia germination (%)

| Category | Term | Estimate | Std_Error | Z_value | EDF | Ref_df | Chi_sq | P_value |
| --- | --- | --- | --- | --- | --- | --- | --- | --- |
| Parametric Coefficients | (Intercept) | 6.130 | 4.334 | 1.414 | - | - | - | 0.157 |
| Smooth Terms | f(Temp,Incub) | - | - | - | 23.406 | 23.770 | 1555.575 | 0*** |
| Smooth Terms | r(Temp,Incub,Replicate) | - | - | - | 60.541 | 75 | 1961.809 | 0*** |
| Model Summary | R-squared (adjusted) | 0.971 | - | - | - | - | - | - |
| Model Summary | Deviance explained | 0.948 | - | - | - | - | - | - |
| Model Summary | -REML | 4378.606 | - | - | - | - | - | - |
| Model Summary | Scale estimation | 1 | - | - | - | - | - | - |
| Model Summary | Sample size | 704 | - | - | - | - | - | - |

Estimate is the effect size; Std_Error is the uncertainty around the estimate; Z_value indicates how far the estimate is from zero; EDF represents the flexibility of smooth terms; Ref_df is the reference degrees of freedom; Chi_sq tests the significance of terms; P_value indicates statistical significance, with values marked as follows: *** for p ≤ 0.001, ** for p ≤ 0.01, * for p ≤ 0.05, . for p ≤ 0.1, and no symbol for p > 0.1.


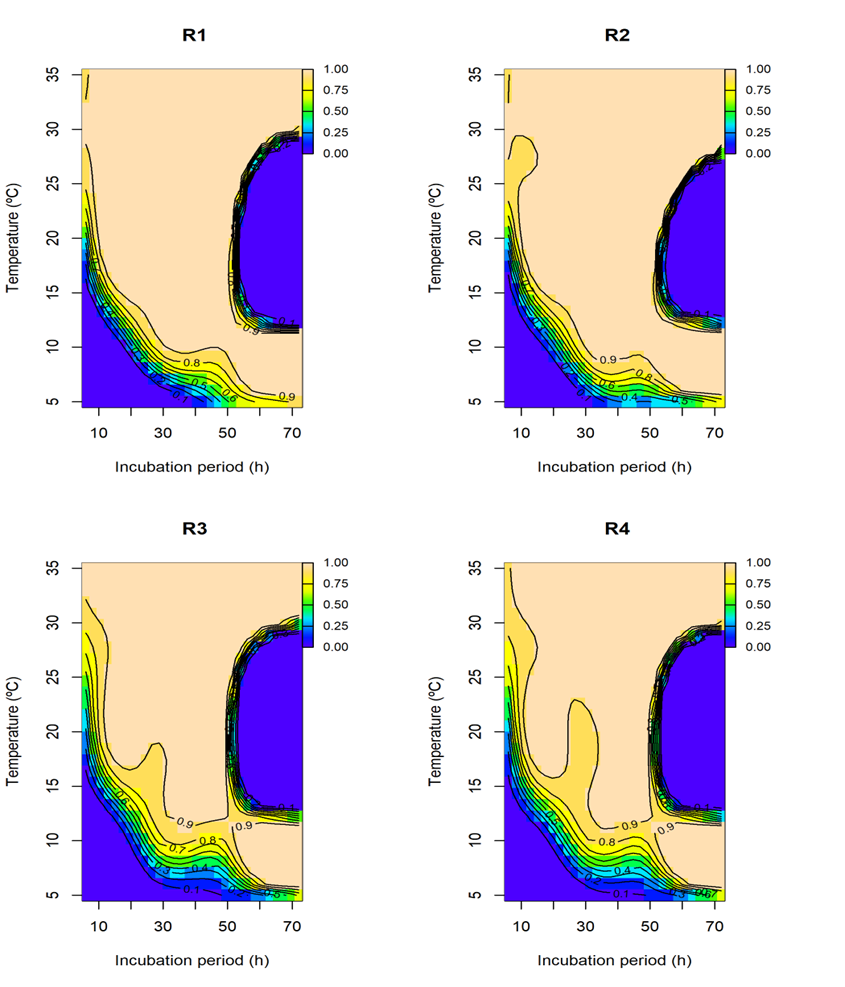


Contour lines connect points with the same predicted value, with warmer colours indicating higher conidia germination and cooler colours representing lower values. Replicate R1 and R2: Isolate PHAL-4. Replicate R3 and R4: isolate PHAL-45.

**Supplementary 14.** Predicted conidia germination (0 to 1 scale) as a function of temperature (°C) and incubation time (h) from the random interaction effect model per replicate


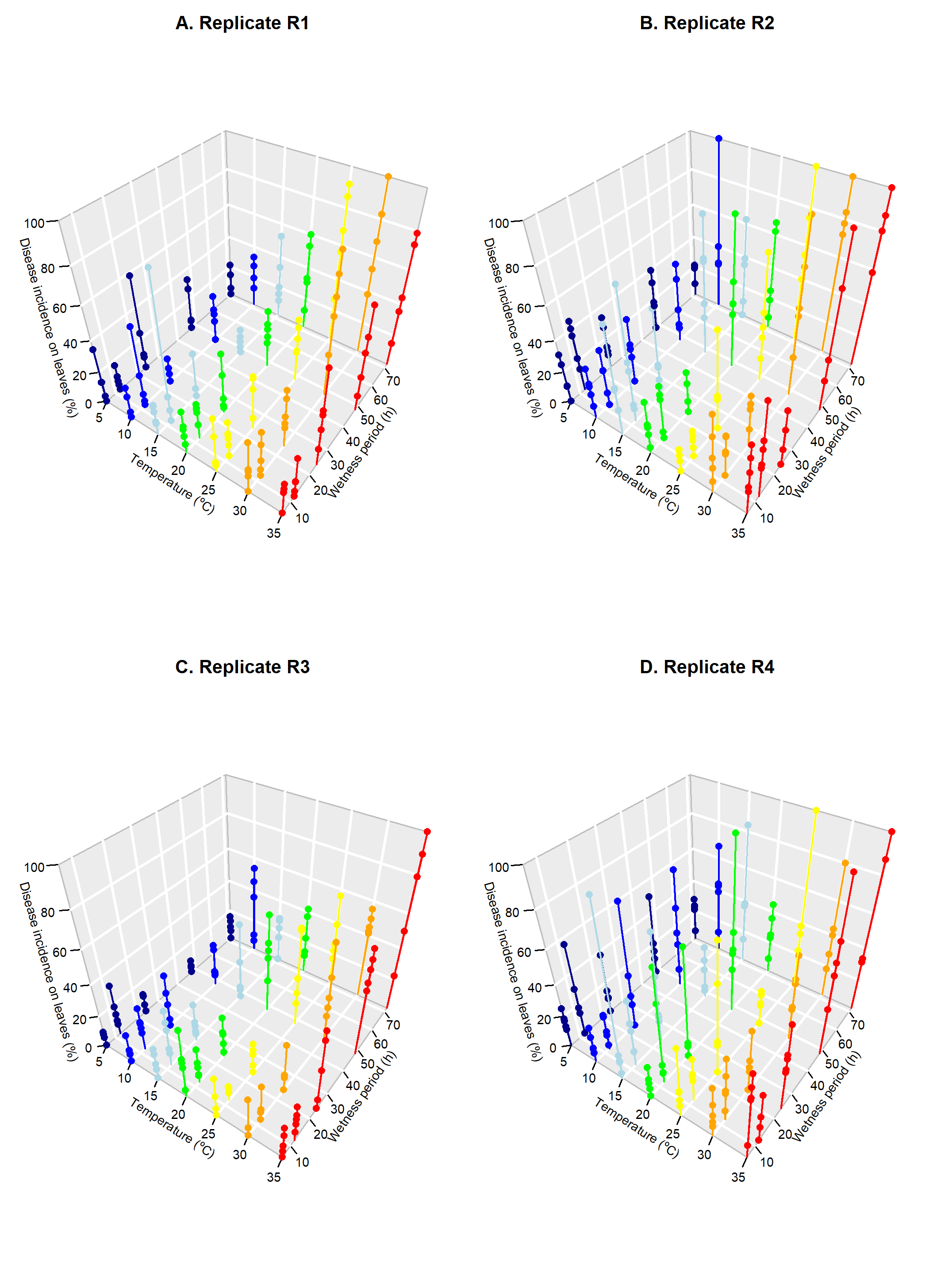


**Supplementary 15.** Exploratory analysis of disease incidence on leaves (%) for each combination of temperature (5, 10, 15, 20, 25, 30, and 35 ºC) and wetness periods (6, 12, 24, 48, and 72 h) per replicate (replicate R1 and R2: isolate PHAL-4, replicate R3 and R4: isolate PHAL-45).


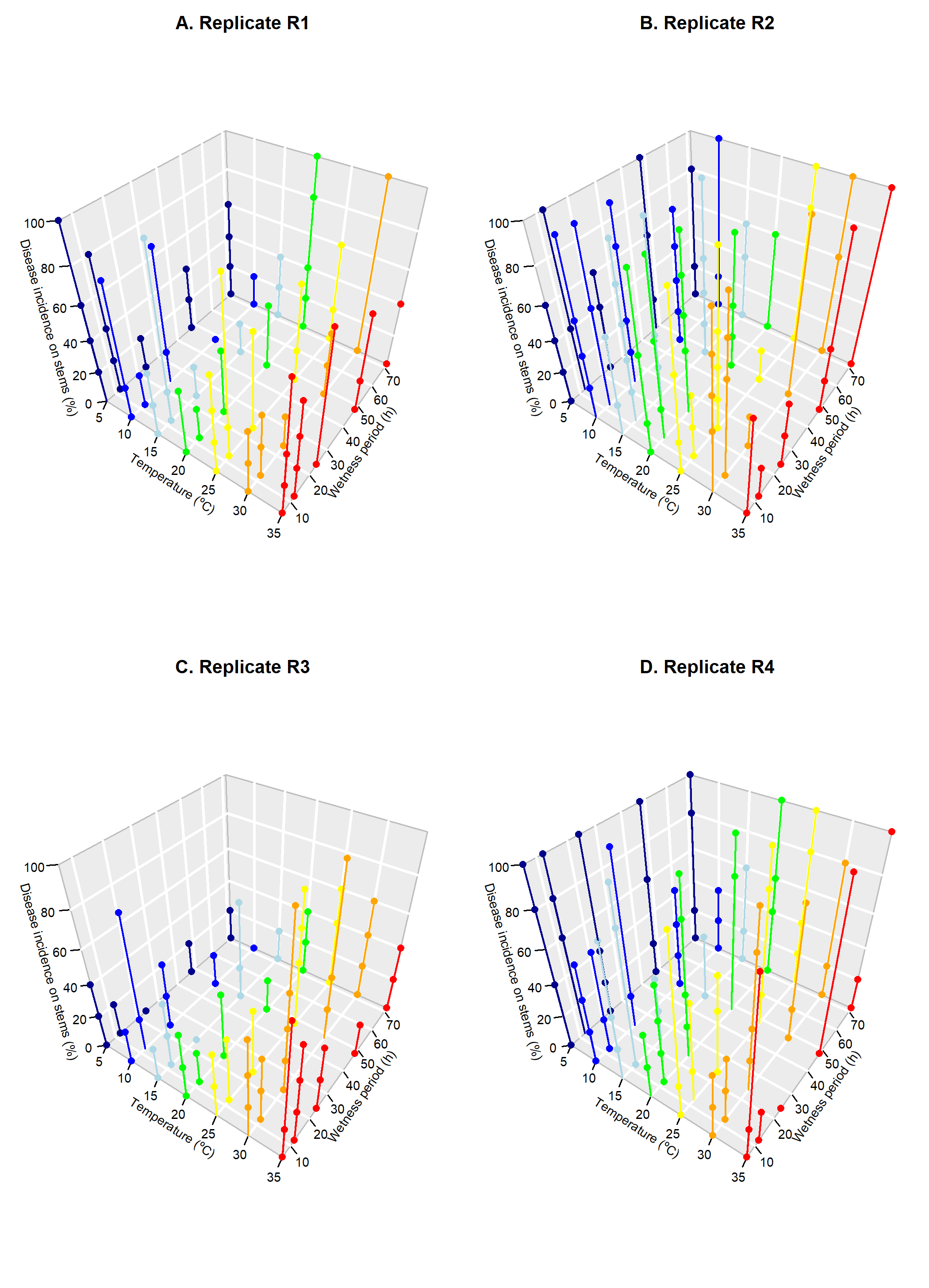


**Supplementary 16.** Exploratory analysis of disease incidence on stems (%) for each combination of temperature (5, 10, 15, 20, 25, 30, and 35 ºC) and wetness periods (6, 12, 24, 48, and 72 h) per replicate

(replicate R1 and R2: isolate PHAL-4, replicate R3 and R4: isolate PHAL-45).

**Supplementary 17.** Comparison and performance in terms of akaike information criterion (AIC), adjusted R², deviance, and the number of parameters of the four statistical models for disease incidence on stems and leaves (%)

| **Disease incidence on leaves (%)** | | | | |  |
| --- | --- | --- | --- | --- | --- |
| Model specification | AIC | R2_adjusted | Deviance | Num_parameters |  |
| Disease incidence on leaves∼  f(Temp,Wetness)^[[14]](#footnote-14)^+  r(Replicate)^[[15]](#footnote-15)^ | 3,717.101 | 0.401 | 0.376 | 19 |  |
| Disease incidence on leaves ∼  f(Temp,Wetness)+  r(Temp,Replicate) ^[[16]](#footnote-16)^+  r(Wetness,Replicate)^[[17]](#footnote-17)^ | 3,749.491 | 0.393 | 0.367 | 23 |  |
| Disease incidence on leaves ∼  f(Temp,Wetness)+  r(Replicate)+  r(Temp,Replicate) +  r(Wetness,Replicate) | 3,712.288 | 0.403 | 0.380 | 27 |  |
| Disease incidence on leaves ∼  f(Temp,Wetness)+  r(Temp,Wetness,Replicate)^[[18]](#footnote-18)^ | **3,609.992** | **0.443** | **0.424** | **60** |  |
| **Disease incidence on stems (%)** | | | | |  |
| Model specification | | AIC | R2_adjusted | Deviance | Num_parameters |
| Disease incidence on stems ∼  f(Temp,Wetness)^[[19]](#footnote-19)^+  r(Replicate)^[[20]](#footnote-20)^ | 2,595.274 | 0.125 | 0.118 | 19 |  |
| Disease incidence on stems ∼  f(Temp,Wetness)+  r(Temp,Replicate)^[[21]](#footnote-21)^+  r(Wetness,Replicate)^[[22]](#footnote-22)^ | 2,660.027 | 0.084 | 0.090 | 23 |  |
| Disease incidence on stems ∼  f(Temp,Wetness)+  r(Replicate)+  r(Temp,Replicate) +  r(Wetness,Replicate) | 2,540.182 | 0.151 | 0.149 | 27 |  |
| Disease incidence on stems ∼  f(Temp,Wetness)+  r(Temp,Wetness,Replicate)^[[23]](#footnote-23)^ | **2,480.116** | **0.191** | **0.192** | **60** |  |

^*^Best fit models are in bold based on the lowest AIC value.

**Supplementary 18.** Summary of the random interaction model for disease incidence on leaves and stems (%)

| Disease incidence on leaves (%) | | | | | | | | |
| --- | --- | --- | --- | --- | --- | --- | --- | --- |
| Category | Term | Estimate | Std_Error | t_value | EDF | Ref_df | F | P_value |
| Parametric Coefficients | (Intercept) | -0.553 | 0.383 | -1.441 | - | - | - | 0.15 |
| Smooth Terms | f(Temp,Wetness) | - | - | - | 8.204 | 8.679 | 80.499 | 0*** |
| Smooth Terms | r(Temp,Wetness,Replicate) | - | - | - | 24.809 | 45 | 296.499 | 0*** |
| Model Summary | R-squared (adjusted) | 0.443 | - | - | - | - | - | - |
| Model Summary | Deviance explained | 0.424 | - | - | - | - | - | - |
| Model Summary | -REML | 1837.768 | - | - | - | - | - | - |
| Model Summary | Scale estimation | 1 | - | - | - | - | - | - |
| Model Summary | Sample size | 697 | - | - | - | - | - | - |
| Disease incidence on stems (%) | | | | | | | | |
| Category | Term | Estimate | Std_Error | t_value | EDF | Ref_df | F | P_value |
| Parametric Coefficients | (Intercept) | -2.206 | 0.64 | -3.447 | - | - | - | 0.001*** |
| Smooth Terms | f(Temp,Wetness) | - | - | - | 7.877 | 9.655 | 39.556 | 0*** |
| Smooth Terms | r(Temp,Wetness,Replicate) | - | - | - | 22.812 | 45 | 276.493 | 0*** |
| Model Summary | R-squared (adjusted) | 0.191 | - | - | - | - | - | - |
| Model Summary | Deviance explained | 0.192 | - | - | - | - | - | - |
| Model Summary | -REML | 1264.514 | - | - | - | - | - | - |
| Model Summary | Scale estimation | 1 | - | - | - | - | - | - |
| Model Summary | Sample size | 697 | - | - | - | - | - | - |

Estimate is the effect size; Std_Error is the uncertainty around the estimate; t_value indicates how far the estimate is from zero; EDF represents the flexibility of smooth terms; Ref_df is the reference degrees of freedom; F tests the significance of terms; P_value indicates statistical significance, with values marked as follows: *** for p ≤ 0.001, ** for p ≤ 0.01, * for p ≤ 0.05, . for p ≤ 0.1, and no symbol for p > 0.1.

**
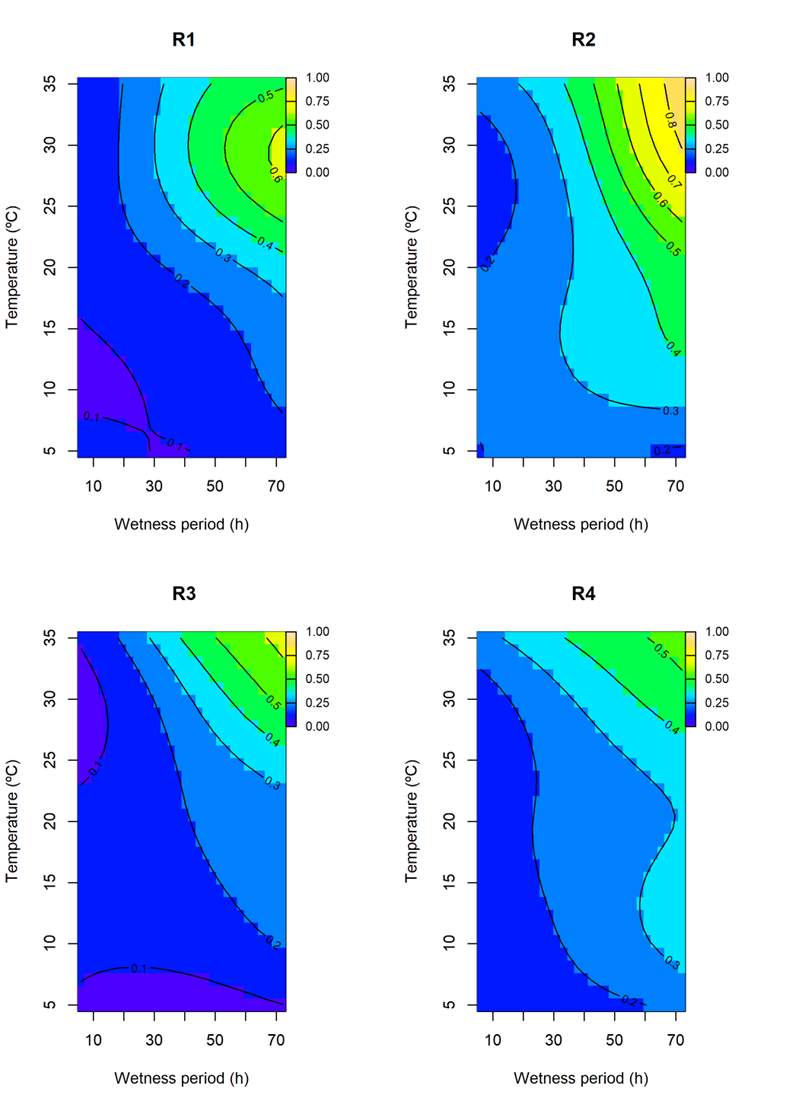
**

Contour lines connect points with the same predicted value, with warmer colours indicating higher disease incidence on leaves and cooler colours representing lower values. Replicate R1 and R2: Isolate PHAL-4. Replicate R3 and R4: isolate PHAL-45.

**Supplementary 19.** Predicted disease incidence on leaves (0-1 scale) as a function of temperature (°C) and wetness period (h) from the random interaction effect model per replicate


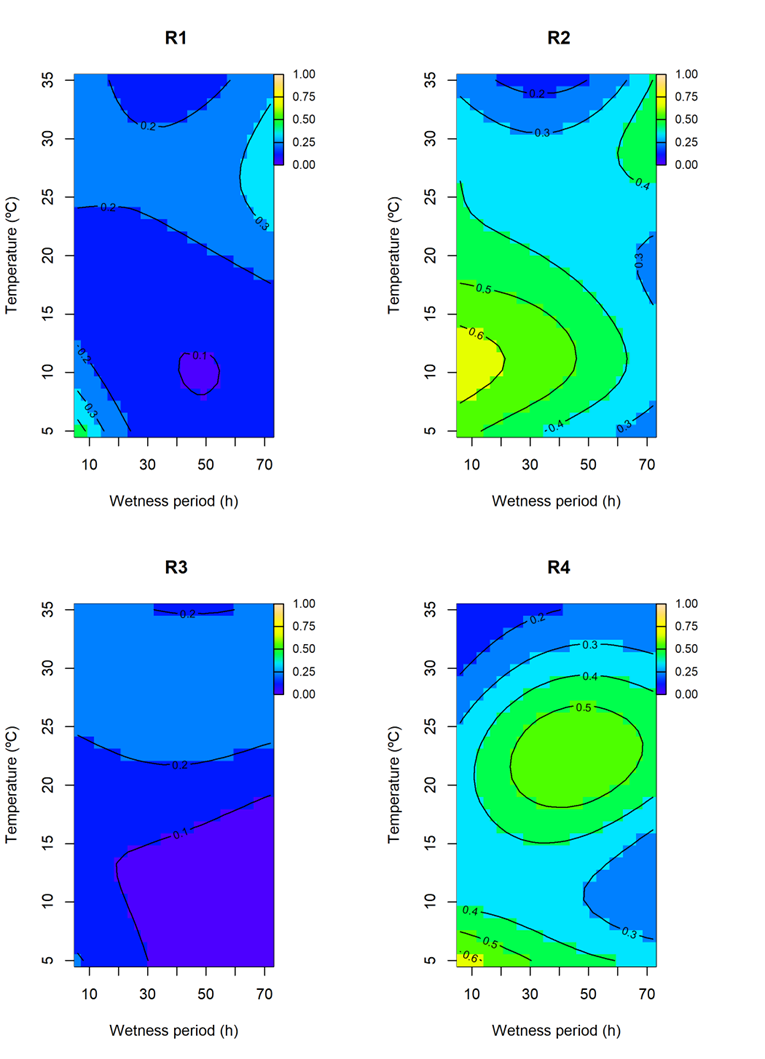


Contour lines connect points with the same predicted value, with warmer colours indicating higher disease incidence on stems and cooler colours representing lower values. Replicate R1 and R2: Isolate PHAL-4. Replicate R3 and R4: isolate PHAL-45.

**Supplementary 20**. Predicted disease incidence on stems (0-1 scale) as a function of temperature (°C) and wetness period (h) from the random interaction model per replicate


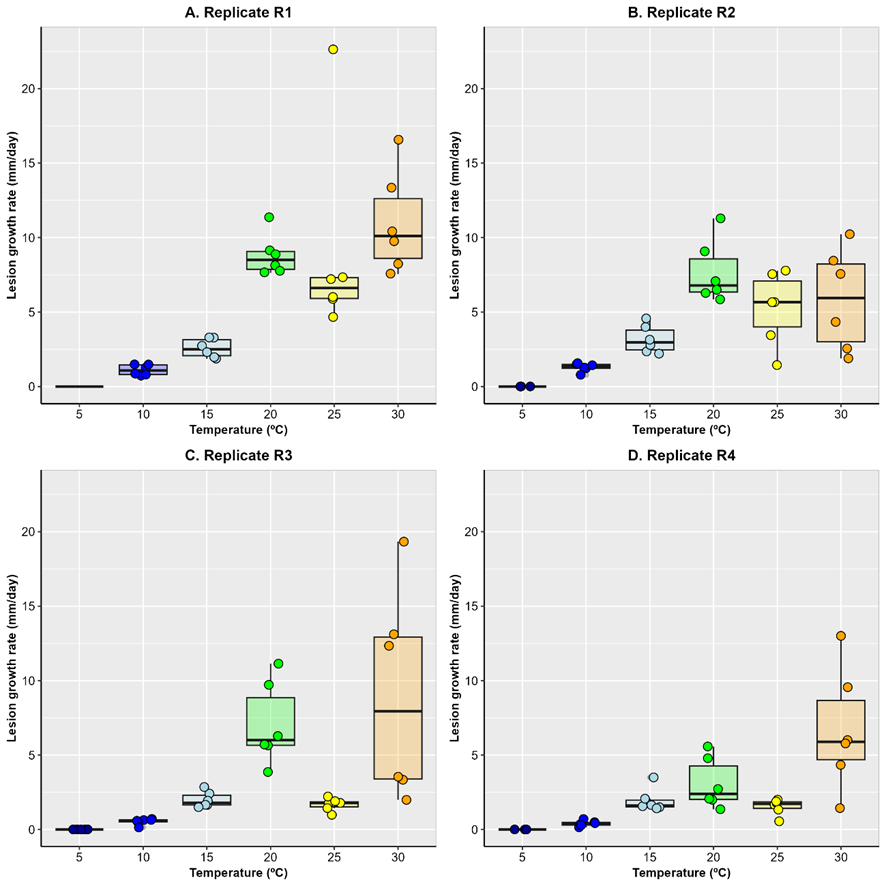


**Supplementary 21**. Exploratory analysis of lesion growth rate (mm day⁻¹) per temperature (5, 10, 15, 20, 25, 30, and 35 ºC) observed per replicate (replicate R1 and R2: isolate PHAL-4, replicate R3 and R4: isolate PHAL-45).

**Supplementary 22**. Comparison and performance in terms of Akaike Information Criterion (AIC), adjusted R², deviance, and the number of parameters of the three statistical models for lesion growth rate (mm day⁻¹)

| Model specification | AIC | R2_adjusted | Deviance | Num_parameters |
| --- | --- | --- | --- | --- |
| Lesion growth rate∼  f(Temp)^[[24]](#footnote-24)^ +  r(Replicate)^[[25]](#footnote-25)^ | 733.437 | 0.480 | 0.493 | 8 |
| Lesion growth rate∼  f(Temp)+  r(Temp,Replicate)^[[26]](#footnote-26)^ | **722.921** | **0.516** | **0.529** | **8** |
| Lesion growth rate∼  f(Temp)+  r(Replicate)+  r(Temp,Replicate) | 722.922 | 0.516 | 0.529 | 12 |

^*^Best fit model is in bold based on the lowest AIC value.

**Supplementary 23.** Summary of the random smooth model for lesion growth rate (mm day⁻¹)

| Category | Term | Estimate | Std_Error | t_value | EDF | Ref_df | F | P_value |
| --- | --- | --- | --- | --- | --- | --- | --- | --- |
| Parametric Coefficients | (Intercept) | 3.732 | 0.719 | 5.189 | - | - | - | 0*** |
| Smooth Terms | f(Temp) | - | - | - | 1 | 1 | 43.458 | 0*** |
| Smooth Terms | r(Temp,Replicate) | - | - | - | 2.719 | 3 | 9.69 | 0*** |
| Model Summary | R-squared (adjusted) | 0.516 | - | - | - | - | - | - |
| Model Summary | Deviance explained | 0.529 | - | - | - | - | - | - |
| Model Summary | -REML | 361.468 | - | - | - | - | - | - |
| Model Summary | Scale estimation | 8.442 | - | - | - | - | - | - |
| Model Summary | Sample size | 144 | - | - | - | - | - | - |

Estimate is the effect size; Std_Error is the uncertainty around the estimate; t_value indicates how far the estimate is from zero; EDF represents the flexibility of smooth terms; Ref_df is the reference degrees of freedom; F tests the significance of terms; P_value indicates statistical significance, with values marked as follows: *** for p ≤ 0.001, ** for p ≤ 0.01, * for p ≤ 0.05, . for p ≤ 0.1, and no symbol for p > 0.1.

1. Smooth function for temperature. [↑](#footnote-ref-1)
2. Random intercept for each experimental replicate. [↑](#footnote-ref-2)
3. Random smooth for temperature by experimental replicate. The smooth function for temperature varies between each experimental replicate. [↑](#footnote-ref-3)
4. Smooth functions for temperature, days post-inoculation, and their interaction. [↑](#footnote-ref-4)
5. Random intercept for each experimental replicate. [↑](#footnote-ref-5)
6. Random smooth for temperature by experimental replicate. The smooth function for temperature varies between each experimental replicate. [↑](#footnote-ref-6)
7. Random smooth for days post-inoculation period by experimental replicate. The smooth function for days post-inoculation varies between each experimental replicate. [↑](#footnote-ref-7)
8. Random interaction for temperature, days post-inoculation, and experimental replicate. [↑](#footnote-ref-8)
9. Smooth functions for temperature, incubation period, and their interaction. [↑](#footnote-ref-9)
10. Random intercept for each experimental replicate. [↑](#footnote-ref-10)
11. Random smooth for temperature by experimental replicate. The smooth function for temperature varies between each experimental replicate. [↑](#footnote-ref-11)
12. Random smooth for incubation period by experimental replicate. The smooth function for incubation period varies between each experimental replicate. [↑](#footnote-ref-12)
13. Random interaction for temperature, incubation period, and experimental replicate. [↑](#footnote-ref-13)
14. Smooth functions for temperature, wetness period, and their interaction. [↑](#footnote-ref-14)
15. Random intercept for each experimental replicate. [↑](#footnote-ref-15)
16. Random smooth for temperature by experimental replicate. The smooth function for temperature varies between each experimental replicate. [↑](#footnote-ref-16)
17. Random smooth for wetness period by experimental replicate. The smooth function for wetness period varies between each experimental replicate. [↑](#footnote-ref-17)
18. Random interaction for temperature, humidity, and experimental replicate. [↑](#footnote-ref-18)
19. Smooth functions for temperature, wetness period, and their interaction. [↑](#footnote-ref-19)
20. Random intercept for each experimental replicate. [↑](#footnote-ref-20)
21. Random smooth for temperature by experimental replicate. The smooth function for temperature varies between each experimental replicate. [↑](#footnote-ref-21)
22. Random smooth for wetness period by experimental replicate. The smooth function for wetness period varies between each experimental replicate. [↑](#footnote-ref-22)
23. Random interaction for temperature, humidity, and experimental replicate. [↑](#footnote-ref-23)
24. Smooth function for temperature. [↑](#footnote-ref-24)
25. Random intercept for each experimental replicate. [↑](#footnote-ref-25)
26. Random smooth for temperature by experimental replicate. The smooth function for temperature varies between each experimental replicate. [↑](#footnote-ref-26)
